# Genetic risk variants for multiple sclerosis are linked to differences in alternative pre-mRNA splicing

**DOI:** 10.3389/fimmu.2022.931831

**Published:** 2022-10-28

**Authors:** Elena Putscher, Michael Hecker, Brit Fitzner, Nina Boxberger, Margit Schwartz, Dirk Koczan, Peter Lorenz, Uwe Klaus Zettl

**Affiliations:** ^1^ Rostock University Medical Center, Department of Neurology, Division of Neuroimmunology, Rostock, Germany; ^2^ Rostock University Medical Center, Institute of Immunology, Rostock, Germany

**Keywords:** B cells, genetic disease risk, splicing reporter minigene assay, multiple sclerosis, single-nucleotide polymorphisms, TSFM, alternative splicing

## Abstract

**Background:**

Multiple sclerosis (MS) is a chronic immune-mediated disease of the central nervous system to which a genetic predisposition contributes. Over 200 genetic regions have been associated with increased disease risk, but the disease-causing variants and their functional impact at the molecular level are mostly poorly defined. We hypothesized that single-nucleotide polymorphisms (SNPs) have an impact on pre-mRNA splicing in MS.

**Methods:**

Our study focused on 10 bioinformatically prioritized SNP–gene pairs, in which the SNP has a high potential to alter alternative splicing events (ASEs). We tested for differential gene expression and differential alternative splicing in B cells from MS patients and healthy controls. We further examined the impact of the SNP genotypes on ASEs and on splice isoform expression levels. Novel genotype-dependent effects on splicing were verified with splicing reporter minigene assays.

**Results:**

We were able to confirm previously described findings regarding the relation of MS-associated SNPs with the ASEs of the pre-mRNAs from *GSDMB* and *SP140*. We also observed an increased *IL7R* exon 6 skipping when comparing relapsing and progressive MS patients to healthy subjects. Moreover, we found evidence that the MS risk alleles of the SNPs rs3851808 (*EFCAB13*), rs1131123 (*HLA-C*), rs10783847 (TSFM), and rs2014886 (*TSFM*) may contribute to a differential splicing pattern. Of particular interest is the genotype-dependent exon skipping of *TSFM* due to the SNP rs2014886. The minor allele T creates a donor splice site, resulting in the expression of the exon 3 and 4 of a short *TSFM* transcript isoform, whereas in the presence of the MS risk allele C, this donor site is absent, and thus the short transcript isoform is not expressed.

**Conclusion:**

In summary, we found that genetic variants from MS risk loci affect pre-mRNA splicing. Our findings substantiate the role of ASEs with respect to the genetics of MS. Further studies on how disease-causing genetic variants may modify the interactions between splicing regulatory sequence elements and RNA-binding proteins can help to deepen our understanding of the genetic susceptibility to MS.

## Introduction

Multiple sclerosis (MS) is a chronic immune-mediated and neurodegenerative disease of the central nervous system (CNS) (1, 2). Approximately 2.8 million people worldwide suffer from MS, with women being affected two to three times more often than men and with an average age at diagnosis of 32 years (3, 4). MS is classified into three different clinical courses: relapsing–remitting MS (RRMS) as the most common form (~85% of initial diagnoses), secondary progressive MS (SPMS), and primary progressive MS (PPMS) (~15% of initial diagnoses) (5–7). Clinically, RRMS is characterized by episodes of disease (relapses) followed by a partial recovery of symptoms (remissions). As the neurological deficits worsen with disease progression, approximately 80% of the RRMS cases convert to SPMS within 25 years after the diagnosis (6, 8, 9). PPMS and SPMS are characterized by a continuous worsening of symptoms without significant recovery. The symptoms of MS include, among others, limited mobility, impaired vision, and cognitive deficits (10). The severity of disability is usually determined by the Expanded Disability Status Scale (EDSS) (11).

The immune system plays a key role in the pathophysiology of MS. Immune cells infiltrate the CNS across the blood–brain barrier, leading to demyelination, neuroaxonal damage, the loss of synapses, and reactive gliosis (1, 8, 12). The disruption of neuronal signal transmission then results in clinical symptoms. Approaches to the management of MS include the treatment of acute relapses (13, 14), symptomatic therapies (15), and therapies to prevent relapses and slow the progression of disability (16–18). B cells are central players in the pathogenesis of MS as they mediate cytokine production, antigen presentation, intrathecal antibody synthesis, and the formation of ectopic follicles (19). As new research findings on MS immunopathology further underlined the functional role of B cells, disease-modifying drugs that mediate the depletion of B cells are now commonly used (20–24).

The etiology of MS is still unclear. However, environmental and lifestyle factors, such as smoking, adolescent obesity, and Epstein–Barr virus (EBV) infection, as well as genetic predisposition have been identified as risk factors contributing to the development of MS (25–29). Single-nucleotide polymorphisms (SNPs), the variations of single base pairs at specific genome locations, are the most common type of genetic risk factors (30, 31). Genome-wide association studies (GWASs) have been used to identify associations between SNP alleles and disease. In the most recent GWAS of MS, 233 SNPs have been associated with a significantly increased risk of developing MS [MS-associated lead SNPs (MS SNPs)] (32). However, considering the tendency of proximal SNPs to be inherited together (33), SNPs that are in linkage disequilibrium (LD) with an MS SNP are also associated with MS. Most disease-associated SNPs are considered to have regulatory implications, which means that they are colocalized with quantitative trait loci (QTLs) and thus can affect, e.g., gene expression (eQTL) or alternative splicing (sQTL) (34–38).

Precursor messenger RNA (pre-mRNA) splicing is a physiological process in the cell nucleus by which the introns (intragenic regions) of a pre-mRNA are cut out and the remaining exons (expressed regions) are joined together to form a mature mRNA molecule (39). The cotranscriptional splicing process is coordinated by a complex interplay of *cis*-elements, *trans*-acting factors, and the spliceosome complex, which consists of five small nuclear ribonucleoproteins (snRNPs) (40, 41). The important sequences within the pre-mRNA are 5’ and 3’ splice sites (donor and acceptor, respectively), the branch point, the polypyrimidine tract, and exonic or intronic motifs to enhance or silence splicing (42–45). The RNA-binding proteins (RBPs) that recognize these sequences are important for the recruitment of the spliceosome complex. The regulation of the splicing process enables the use of different splice sites, which, in turn, leads to alternative splicing and thus to an altered exon usage compared to the canonical splicing. This allows for the generation of various mRNAs from one pre-mRNA, resulting in a broad transcriptome diversity.

There are five basic types of alternative splicing events (ASEs). While during *exon skipping*, an exon is excised and not inserted into the mRNA, during *intron retention*, an intron is not removed and remains in the mRNA molecule. The use of different splice sites can also result in *mutually exclusive exons*, where only one of two possible exons occurs in the mRNA, or in exons with different lengths due to the use of different *acceptor* or *donor splice sites* (46). In addition to the physiological role of alternative splice sites, genetic variants, such as SNPs, can alter the splicing pattern and thereby contribute to the risk of developing diseases (47). As the majority of ASEs in the human EST database are not conserved in mice (48), investigations on the splicing pattern in the experimental autoimmune encephalomyelitis (EAE) mouse model for MS are limited, and thus studies with MS patients are needed. We previously reviewed studies in which ASEs in association with MS have been investigated and found that alternative splicing in MS has been little studied so far (49). The most prominent example is exon 6 skipping in the transcript for the interleukin-7 receptor (*IL7R*) dependent on SNP rs6897932 (50).

In this study, we investigated ASEs related to SNPs in genetic loci associated with the risk of MS. For this purpose, we used a bioinformatic approach to identify SNPs that potentially alter splicing in MS. We then measured the expression of genes and of individual exons and exon–exon junctions in B cells from MS patients and healthy individuals and analyzed whether the expression is related to MS and/or the SNP. We further used splicing reporter minigene assays to verify alternative pre-mRNA splicing dependent on the genotype of the SNPs. Our study provides new insights into the molecular pathomechanisms of MS by exploring the putative functional role of genetic variants associated with disease susceptibility.

## Methods

**Figure 1:**
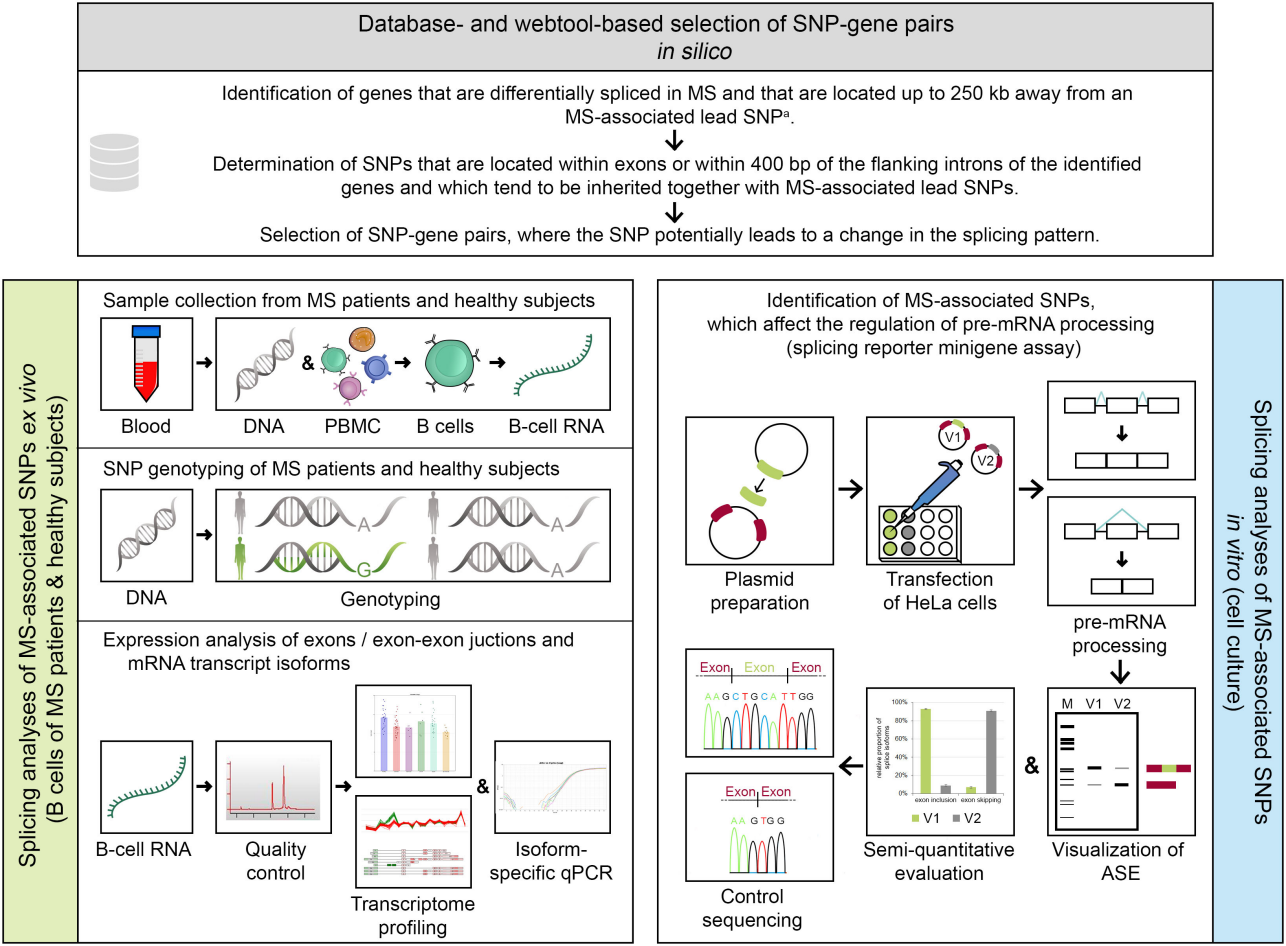
Methodical overview of the study. An *in silico* approach (serial workflow) was employed to identify single-nucleotide polymorphism (SNP)–gene pairs, where the SNP has the potential to alter the splicing pattern of a gene. For the selected SNP–gene pairs, *ex vivo* and *in vitro* analyses were conducted. ^a^ Multiple sclerosis (MS)–associated lead SNP according to the genome-wide association study (GWAS) of MS from 2019 (32). ASE, alternative splicing event; bp, base pairs; GWAS, genome-wide association study; kb, kilobases; MS, multiple sclerosis; PBMC, peripheral blood mononuclear cells; qPCR, quantitative real-time PCR polymerase chain reaction; SNP, single-nucleotide polymorphism; V1/V2, minigene construct variants, which differ in only one base and thus represent the two allelic variants of a SNP (here, V1 shows constitutive splicing and V2 shows alternative splicing).

This study is divided into *in silico*, *ex vivo*, and *in vitro* parts (**Figure 1**). A detailed description of all methods is provided in the supplement (**Supplementary file**).

### Selection of multiple sclerosis–associated genetic variants that may alter pre-mRNA splicing

Using publicly available microarray data sets and a literature-based screening, we identified differentially spliced candidate genes in MS that are encoded less than 250 kb away from an MS SNP (32). We then determined SNPs that are at least in mild LD (*r* ^2^ > 0.1 and *D’* > 0.7) with the MS SNPs and are located within exons or adjacent intronic regions (up to 400 bp from the exon) of the genes. By using the splice prediction tool Human Splicing Finder (51) and the POSTAR2 database (52), we finally prioritized 10 SNP–gene pairs, in which the SNP has the potential to alter the splicing pattern of the gene (hereafter referred to as splice SNP).

### Study cohort

As part of the research projects of the Neuroimmunology research group at Rostock University Medical Center, a total of 121 blood samples were collected and DNA and B-cell RNA were extracted as described previously (53). The subjects were divided into the following three subgroups: healthy controls, PPMS patients, and RRMS patients. MS patients were diagnosed according to the 2017 revisions of the McDonald criteria (54). The diagnosis, treatment, and monitoring of the patients followed routine clinical practice. For further details on the study cohort and the B-cell samples, the reader is referred to our previously published study (53).

### Single-nucleotide polymorphism genotyping

The genotyping of the 10 bioinformatically determined splice SNPs was performed with the DNA extracted from the blood samples. For the genotyping, we used custom TaqMan^®^ Array Cards (Applied Biosystems). Data analysis was performed in an automated manner using the TaqMan Genotyper Software (version 1.6, Applied Biosystems). The genotype assignments were manually validated. In case of failed genotyping, the SNP was not considered for further analyses.

### Transcriptome analysis

The isolated B-cell RNA samples were used to perform high-density microarray measurements. This was done with Clariom D arrays for human (Thermo Fisher Scientific), which allow to examine the expression of more than 130,000 protein-coding and non-protein-coding genes (transcript clusters, TC probe sets). The arrays are designed using six oligonucleotide probes for each probe selection region (PSR), mostly identical with an exon, and four probes for all presumptive exon–exon junctions (junction probe set, JUC), which enables the analysis of expression differences with respect to single exons or exon–exon junctions. Sample preparation and microarray hybridization were conducted as described in the **supplementary methods (Supplementary file)**. Based on the transcriptome data for all 121 samples, we tested for differential gene expression and splicing pattern differences in MS patients *vs*. healthy controls as well as in the dependence of the splice SNP genotypes. The analysis of the microarray data was accomplished by using the Transcriptome Analysis Console (TAC) software (version 4.0.2, Applied Biosystems).

### Verification of splice isoform expression *via* quantitative real-time PCR

After the transcriptome analysis, sufficient material was available for 109 of the 121 B-cell RNA samples to perform transcript isoform expression measurements by quantitative (real-time) PCR (qPCR) assays. Custom TaqMan^®^ Gene Expression Array Cards (Thermo Fisher Scientific) were used for this analysis. For each of the 10 SNP–gene pairs, two qPCR assays were used to distinguish the different transcript isoforms resulting from the specific ASEs under scrutiny (e.g., exon skipping *vs*. exon inclusion). If a transcript isoform could not be detected within 45 PCR cycles, the missing C_T_ values were imputed with the R package nondetects (55). Primary data analysis was conducted by using the ExpressionSuite software (version 1.3, Thermo Fisher Scientific). The data were normalized and converted to the linear scale (**Supplementary file**).

### Splicing reporter minigene assay

Seven SNP–gene pairs were subjected to splicing reporter minigene assays. The minigene assay is based on the principle of the transient transfection of cells with a vector containing the genomic region of interest cloned between two constitutive exons. Our minigene constructs were generated using the pDESTsplice vector and synthesized genomic sequences cloned into the pDONR221 vector (BioCat). The pDESTsplice vector was kindly gifted by Stefan Stamm (56) (Addgene plasmid #32484; http://n2t.net/addgene:32484; RRID: Addgene_32484). For each SNP–gene pair, our minigene assays always consisted of two minigene constructs that differed in a single base and thus represented the two SNP allele variants. HeLa cells were transiently transfected with the minigene constructs. RNA from the HeLa cells was isolated 24 h after the transfection and used for RT-PCR. The PCR products were visualized by gel electrophoresis and validated by sequencing. The distribution of splice isoforms was evaluated by determining the intensity of the PCR product bands on the gel with the Image Studio Lite software version 5.2 (LI-COR Biosciences).

### Statistics

Statistical analyses were performed in R (version 4.0) and the TAC software (version 4.0.2). For descriptive statistics, the (robust) means and standard deviations (SD) per group were either calculated in R or directly obtained from the TAC software. We computed linear models and performed pairwise comparisons with Tukey *post-hoc* tests by using either the limma (57) framework in TAC or the R packages car (58) and stats. For the evaluation of the minigene assay outcomes, we performed two-way analyses of variance (ANOVA) to test whether the relative transcript abundance can be explained by an interaction between the splice SNP allele and the splice isoform. For all analyses, a significance level of α = 0.01 was chosen to indicate significant differences in expression and splicing, respectively. This cutoff was chosen to provide a balance between multiple testing and exploratory investigations. The data were visualized with bar plots and beeswarm plots.

## Results

### Prioritization of splice single-nucleotide polymorphisms in multiple sclerosis–associated genetic loci

We identified a total of 10 SNP–gene pairs in which the splice SNP has the potential to influence pre-mRNA splicing and for which we sought an experimental validation of the determined ASEs in this work (**Table 1**; **Figure S1**). For three SNP–gene pairs (genes: *GSDMB*, *IL7R*, and *SP140*), an aberrant alternative splicing in MS has already been described in the literature (49).

**Table 1:** Prioritized SNPs from MS–associated genetic regions that potentially alter the splicing pattern of eight genes.

Gene	MS SNP identifier	Splice SNP identifier	Splice SNP position^b^	Alleles splice SNP^c^	Global allele frequency splice SNP	MS RA	LD (EUR)		Exon (Ensembl transcript ID)	Dist. splice SNP to exon (bp)^b^	Splicing motif^d^	ASE
							r^2^	D’				
*CLEC16A*	rs2286974	rs11074944	chr16:11003696	G/A	G: 91.15%; A: 8.85%	G	0.10	1.00	exon 11 (ENST00000409790)	+ 391 (3’)	ISE/ISS	alt. 5’ donor site
*CLEC16A*	rs6498163	rs3214361	chr16:11125905	C/-	C: 60.88%; -: 39.12%	C	0.17	0.83	exon 22 (ENST00000409790)	- 74 (5’)	branch point	alt. last exon
*EFCAB13*	rs11079784	rs3851808	chr17:47347778	C/T	T: 61.41%; C: 38.59%	C	0.55	0.98	exon 9 and 10 (ENST00000331493)	- 30 (5’, exon 9)	branch point	exon skipping
*GSDMB* ^a^	rs9909593	rs11078928	chr17:39908216	T/C	T: 62.74%; C: 37.26%	C	0.90	0.98	exon 6 (ENST00000418519)	- 2 (5’)	acceptor site	exon skipping
*HLA-C*	rs9266629	rs1131123	chr6:31271601	G/T	G: 51.57%; T: 48.43%	T	0.13	0.71	exons 2-3 (ENST00000640219)	0 (exon 3)	donor site, ESE/ESS	intron retention
*IL7R* ^a^	rs10063294	rs6897932	chr5:35874473	C/T	C: 76.97%; T: 23.03%	C	0.30	1.00	exon 6 (ENST00000303115)	0	ESE/ESS	exon skipping
*NCAPH2*	rs140522	rs2782	chr22:50523425	C/T	C: 66.04%; T: 33.96%	T	0.75	0.99	exons 19-20 (ENST00000299821)	0 (exon 20)	ESE/ESS	intron retention, alt. last exon
*SP140* ^a^	rs35540610	rs28445040	chr2:230245867	C/T	C: 85.07%; T: 14.93%	T	0.73	0.99	exon 7 (ENST00000420434)	0	ESE/ESS	exon skipping
*TSFM*	rs701006	rs2014886	chr12:57783654	C/T	C: 59.61%; T: 40.39%	C	0.62	0.93	exons 3 and 4 (ENST00000417094)	+ 2 (3’, exon 3)	donor site	exon skipping
*TSFM*	rs701006	rs10783847	chr12:57802664	G/A	G: 55.36%; A: 44.64%	G	0.62	0.92	exons 6 and 7 (ENST00000550559)	0 (exon 7)	ESE/ESS	exon skipping, alt. last exon

Ten SNPs (splice SNPs) that are in LD with nine MS SNPs from the latest GWAS (32) were identified. Those splice SNPs are located in exons or in the adjacent intronic sequences of eight genes and are suspected to alter the splicing pattern. According to splice prediction algorithms, databases, and the existing literature, the splice SNPs potentially lead to the alterations of the branch point, an ESE/ESS, an ISE/ISS, an acceptor splice site, or a donor splice site (splicing motif). We identified four different types of ASEs: alt. 5’ donor site (n = 1), alt. last exon (n = 3), exon skipping (n = 6), and intron retention (n = 2). The allele distribution according to dbSNP build 151 and the splice SNP allele correlating with the MS risk allele of the MS SNP are indicated (MS RA). ^a^ For 3 of the 10 SNP–gene pairs, alternative splicing in MS has already been described in the literature (49). ^b^ Distances and positions according to the GRCh38 reference genome assembly. ^c^ Allele variant annotation for the + strand of the reference genome. ^d^ It is usually difficult to distinguish whether a genetic variant weakens a splicing enhancer or augments a splicing silencer. alt., alternative; ASE, alternative splicing event; bp, base pairs; dist., distance; ESE, exonic splicing enhancer; ESS, exonic splicing silencer; EUR, European population; GWAS, genome-wide association study; ISE, intronic splicing enhancer; ISS, intronic splicing silencer; LD, linkage disequilibrium; MS, multiple sclerosis; MS SNP, MS-associated lead single-nucleotide polymorphism; r^2^ and D’; the measures of LD between MS SNP and splice SNP; SNP, single-nucleotide polymorphism.

The splice SNPs are located within an exonic region (n = 5) or within 400 bp of the adjacent intronic regions (n = 5), with all but one of the intronic SNPs being located less than 100 bp from the exon. Two of the 10 splice SNPs are in complete LD (*D’* = 1) with the MS SNP (32), implying that one SNP allele is always inherited together with one specific MS SNP allele.

In total, we determined four different types of ASEs for the 10 SNP–gene pairs. In most cases, exon skipping was found (n = 6). Moreover, we identified the ASEs intron retention (n = 2), alternative 5’ donor site (n = 1), and alternative last exon (n = 3). Note that in two cases (*TSFM* exon 6 and 7 skipping and *NCAPH2* intron 19 retention), the ASE coincided with the usage of an alternative last exon.

### Characteristics of the study cohort groups

A total of 121 blood samples were collected. We obtained 28 samples from healthy controls, 13 samples from PPMS patients, and 80 samples from RRMS patients. The PPMS patients were treated with glucocorticoids. The RRMS samples were taken from patients receiving alemtuzumab (n = 38), natalizumab (n = 29), cladribine (n = 6), fingolimod (n = 3), glatiramer acetate (n = 3), or interferon beta-1b (n = 1).

The sex ratio was relatively balanced in the PPMS group, whereas there was a non-significant preponderance of women in the healthy control group and the RRMS group (**Table 2**). In terms of age, the healthy controls, with an average age of 28.0 years, were significantly younger than the MS patients (mean age: PPMS: 58.7 years, RRMS: 36.1 years, p < 0.001). The mean disease duration was similar for PPMS patients and RRMS patients. RRMS patients had an average of 0.4 relapses in the year prior to the blood collection and a mean EDSS score of 2.7. PPMS patients had a considerably higher degree of disability, with an average EDSS score of 4.9 (p < 0.001). There were no major imbalances in the demographic and clinical data between the SNP genotype groups (**Supplementary Table S8**, **Supplementary file**).

**Table 2:** Basic information on the study cohort.

Group	Samples (n)	Female (n)	Male (n)	Age in years, mean ± SD	Disease duration in years, mean ± SD	EDSS score, mean ± SD (MV)	Relapses in previous year, mean ± SD
Healthy subjects	28	17	11	28.0 ± 8.9	—	—	—
PPMS patients	13	5	8	58.7 ± 9.8	9.7 ± 4.6	4.9 ± 1.7	0.0 ± 0.0
RRMS patients	80	57	23	36.1 ± 10.6	8.0 ± 6.9	2.7 ± 1.3 (10)	0.4 ± 0.7

In this study, a total of 121 blood samples were analyzed. Demographic and clinical data were recorded at the time of blood collection. For 10 samples, no information was available on the patients’ current degree of disability as rated by the Expanded Disability Status Scale (11). —, not available; EDSS, Expanded Disability Status Scale; MV, missing values; n, number; PPMS, primary progressive multiple sclerosis; RRMS, relapsing–remitting multiple sclerosis; SD, standard deviation.

### Differential gene expression and alternative splicing in B cells

The transcriptome data for the 121 B-cell RNA samples were used to test the prioritized genes for differential gene expression and differential alternative splicing. Comparing the gene expression between the study groups, we found a significantly lower *IL7R* mRNA expression in MS patients as compared to healthy controls (**Table 3**). For two genes, we observed a significant association with the splice SNP genotype. The transcript levels of *EFCAB13* were significantly higher when the MS risk allele C of splice SNP rs3851808 was present. For *GSDMB*, a significantly lower gene expression was observed in the homozygous carriers of the MS risk allele C of splice SNP rs11078928.

**Table 3:** Differential gene expression in the B-cell transcriptome data set.

Gene (transcript cluster)	MS patients vs. healthy controls			Splice SNP	Genotypes		
	Group (n)	Mean ± SD	p-value		RA (n)	Mean ± SD	p-value
*CLEC16A* (TC1600006893.hg.1)	Healthy (n = 28)	9.14 ± 0.46	0.3654	rs11074944	2 RA (n = 110)	9.19 ± 0.47	0.8570
	PPMS (n = 13)	9.38 ± 0.56			1 RA (n = 11)	9.06 ± 0.67	
	RRMS (n = 80)	9.19 ± 0.48			0 RA (n = 0)	—	
				rs3214361	genotyping failed		
*EFCAB13* (TC1700012275.hg.1)	Healthy (n = 28)	9.21 ± 0.64	0.7842	rs3851808	2 RA (n = 22)	9.69 ± 0.55	**0.0007**
	PPMS (n = 13)	9.52 ± 0.87		1 RA (n = 53)	9.36 ± 0.61		
	RRMS (n = 80)	9.31 ± 0.55		0 RA (n = 46)	9.09 ± 0.55		
*GSDMB* (TC1700010590.hg.1)	Healthy (n = 28)	7.99 ± 0.57	0.3921	rs11078928	2 RA (n = 12)	6.63 ± 1.24	**2.6e-06**
	PPMS (n = 13)	8.67 ± 0.96		1 RA (n = 71)	8.23 ± 0.86		
	RRMS (n = 80)	8.03 ± 1.27		0 RA (n = 38)	8.24 ± 1.20		
*HLA-C* (TC0600014257.hg.1)	Healthy (n = 28)	15.09 ± 0.85	0.6173	rs1131123*	2 RA (n = 33)	15.25 ± 0.86	0.2320
	PPMS (n = 13)	15.15 ± 0.83		1 RA (n = 73)	15.53 ± 0.91		
	RRMS (n = 80)	15.52 ± 0.91		0 RA (n = 15)	15.05 ± 0.75		
*IL7R* (TC0500007138.hg.1)	Healthy (n = 28)	11.28 ± 1.86	**2.9e-06**	rs6897932	2 RA (n = 70)	9.86 ± 2.14	0.9673
	PPMS (n = 13)	8.19 ± 0.84		1 RA (n = 41)	9.79 ± 2.02		
	RRMS (n = 80)	9.60 ± 1.94		0 RA (n = 10)	9.93 ± 1.08		
*NCAPH2* (TC2200007811.hg.1)	Healthy (n = 28)	6.13 ± 0.32	0.8099	rs2782	2 RA (n = 21)	6.16 ± 0.38	0.5645
	PPMS (n = 13)	6.08 ± 0.36		1 RA (n = 65)	6.17 ± 0.40		
	RRMS (n = 80)	6.20 ± 0.45		0 RA (n = 35)	6.16 ± 0.46		
*SP140* (TC0200011020.hg.1)	Healthy (n = 28)	14.48 ± 0.41	0.0178	rs28445040	2 RA (n = 6)	14.79 ± 0.43	0.9303
	PPMS (n = 13)	14.83 ± 0.70		1 RA (n = 48)	14.79 ± 0.54		
	RRMS (n = 80)	14.85 ± 0.51		0 RA (n = 67)	14.76 ± 0.54		
*TSFM* (TC1200012654.hg.1)	Healthy (n = 28)	5.64 ± 0.34	0.1090	rs2014886*	2 RA (n = 59)	5.75 ± 0.33	0.4091
	PPMS (n = 13)	5.62 ± 0.28		1 RA (n = 51)	5.62 ± 0.36		
	RRMS (n = 80)	5.72 ± 0.34		0 RA (n = 11)	5.61 ± 0.32		
	rs10783847			2 RA (n = 60)	5.75 ± 0.33	0.3408
	1 RA (n = 50)			5.61 ± 0.36		
	0 RA (n = 11)			5.61 ± 0.32		

The expression of the eight prioritized genes in B cells from peripheral blood is reported as Tukey biweight means and standard deviations of log2 signal intensities per group (mean ± SD).A total of 121 samples were analyzed. The numbers of samples according to the study group and splice SNP genotype are given in brackets. Significant expression differences (p < 0.01) are shown in bold. We observed significantly lower mRNA levels in patients with MS as compared to healthy controls for IL7R. For EFCAB13 and GSDMB, we saw a genotype-dependent gene expression. * For technical reasons, the designated splice SNP was tagged by a proximal SNP (Supplementary file). —, not available; MS, multiple sclerosis; n, number; PPMS, primary progressive MS; RA, risk allele; RRMS, relapsing–remitting MS; SNP, single-nucleotide polymorphism.

Next, we used the transcriptome data set to examine differences in the expression levels of individual exons and exon–exon junctions that distinguish certain alternative pre-mRNA splice variants. For this purpose, the data for PSR and JUC probe sets, which correspond to the ASEs of the 10 prioritized SNP–gene pairs, were compared between the study groups and the splice SNP genotypes. When the MS patients were compared with the healthy controls, an evidence of differential splicing was found for three genes (**Table 4**). For the probe set interrogating the exon 6 of *IL7R*, we found significantly higher levels in the healthy group, suggesting that in those individuals, the exon is frequently incorporated into the mRNA. Similarly, we measured significantly higher levels for the probe set corresponding to exon 4 of *TSFM* in healthy controls as compared to patients with MS. In addition, we found that the longer *CLEC16A* exon 11, which belongs to the ENST00000409790 transcript variant, was significantly more abundant in the B cells of MS patients (especially PPMS patients) than in those of healthy controls.

**Table 4:** Differential alternative splicing in the B-cell transcriptome data set.

Gene (PSR/JUC^a^)	MS patients *vs*. healthy controls			Splice SNP	Genotypes		
	Group (n)	Mean ± SD	p-value		RA (n)	Mean ± SD	p-value
*CLEC16A* (PSR1600149031.hg.1, long exon 11)	Healthy (n = 28)	9.65 ± 0.29	**0.0055**	rs11074944	2 RA (n = 110)	9.86 ± 0.49	0.9622
	PPMS (n = 13)	10.03 ± 0.28		1 RA (n = 11)	10.04 ± 0.35		
	RRMS (n = 80)	9.96 ± 0.52		0 RA (n = 0)	—		
*CLEC16A* (PSR1600149066.hg.1, exon 22)	Healthy (n = 28)	7.62 ± 0.53	0.3295	rs3214361	genotyping failed		
	PPMS (n = 13)	7.85 ± 0.57					
	RRMS (n = 80)	7.79 ± 0.62					
*EFCAB13* (JUC1700073491.hg.1, exon 9 to exon 10 junction)	Healthy (n = 28)	4.74 ± 1.25	0.5450	rs3851808	2 RA (n = 22)	6.73 ± 0.97	**5.8e-23**
	PPMS (n = 13)	5.21 ± 1.75		1 RA (n = 53)	5.01 ± 1.38		
	RRMS (n = 80)	3.96 ± 1.68		0 RA (n = 46)	3.13 ± 0.48		
*GSDMB* (PSR1700183459.hg.1, exon 6)	Healthy (n = 28)	9.91 ± 0.89	0.3003	rs11078928	2 RA (n = 12)	7.94 ± 0.97	**1.2e-09**
	PPMS (n = 13)	10.91 ± 1.11		1 RA (n = 71)	10.13 ± 1.04		
	RRMS (n = 80)	9.97 ± 1.54		0 RA (n = 38)	10.47 ± 1.44		
*HLA-C* (PSR0600200977.hg.1, exons 2 and 3 with intron 2)	Healthy (n = 28)	15.90 ± 0.79	0.6744	rs1131123*	2 RA (n = 33)	16.09 ± 0.70	**5.8e-06**
	PPMS (n = 13)	15.87 ± 0.53		1 RA (n = 73)	15.85 ± 0.68		
	RRMS (n = 80)	15.84 ± 0.83		0 RA (n = 15)	14.93 ± 0.93		
*IL7R* (PSR0500148308.hg.1, exon 6)	Healthy (n = 28)	10.50 ± 1.83	**4.7e-05**	rs6897932	2 RA (n = 70)	9.69 ± 1.89	0.2265
	PPMS (n = 13)	8.10 ± 0.65		1 RA (n = 41)	9.04 ± 1.90		
	RRMS (n = 80)	9.22 ± 1.77		0 RA (n = 10)	9.00 ± 0.58		
*NCAPH2* (JUC2200052281.hg.1, exon 19 to exon 20 junction)	Healthy (n = 28)	5.44 ± 0.43	0.3229	rs2782	2 RA (n = 21)	5.52 ± 0.66	0.7605
	PPMS (n = 13)	5.69 ± 0.79		1 RA (n = 65)	5.42 ± 0.56		
	RRMS (n = 80)	5.46 ± 0.65		0 RA (n = 35)	5.56 ± 0.73		
*SP140* (JUC0200064656.hg.1, exon 6 to exon 8 junction)	Healthy (n = 28)	8.62 ± 1.25	0.4886	rs28445040	2 RA (n = 6)	11.24 ± 0.61	**1.4e-31**
	PPMS (n = 13)	9.25 ± 1.19		1 RA (n = 48)	9.78 ± 0.68		
	RRMS (n = 80)	8.77 ± 1.24		0 RA (n = 67)	7.97 ± 0.70		
*TSFM* (PSR1200200788.hg.1, exon 4)	Healthy (n = 28)	5.97 ± 0.55	**0.0066**	rs2014886*	2 RA (n = 59)	5.46 ± 0.50	**5.0e-07**
	PPMS (n = 13)	5.82 ± 0.58		1 RA (n = 51)	5.78 ± 0.53		
	RRMS (n = 80)	5.57 ± 0.56		0 RA (n = 11)	6.65 ± 0.54		
*TSFM* (PSR1200200803.hg.1, exon 7)	Healthy (n = 28)	3.26 ± 0.40	0.8234	rs10783847	2 RA (n = 60)	3.33 ± 0.45	**0.0011**
	PPMS (n = 13)	3.37 ± 0.49		1 RA (n = 50)	3.15 ± 0.38	
	RRMS (n = 80)	3.20 ± 0.43		0 RA (n = 11)	2.85 ± 0.28	

The expression of specific exons and exon–exon junctions in B cells from the peripheral blood was analyzed for the ASEs of the 10 SNP–gene pairs. Tukey biweight means and standard deviations of log2 signal intensities are reported per group (mean ± SD). Data from a total of 121 samples were analyzed, with the number of samples per study group and splice SNP genotype given in brackets. Significant expression differences (p < 0.01) are shown in bold. The data indicated genotype-dependent pre-mRNA splicing for six SNP–gene pairs. ^*^ For technical reasons, the designated splice SNP was tagged by a proximal SNP (**Supplementary file**). ^a^ Summary statistics for all ASE specific PSR JUCs are provided in **Supplementary Tables S9** and **S10** (**Supplementary file**). —, not available; ASE, alternative splicing event; JUC, junction probe set; MS, multiple sclerosis; n, number; PPMS, primary progressive MS; PSR, probe selection region; RA, risk allele; RRMS, relapsing–remitting MS; SNP, single-nucleotide polymorphism.

**Figure 2:**
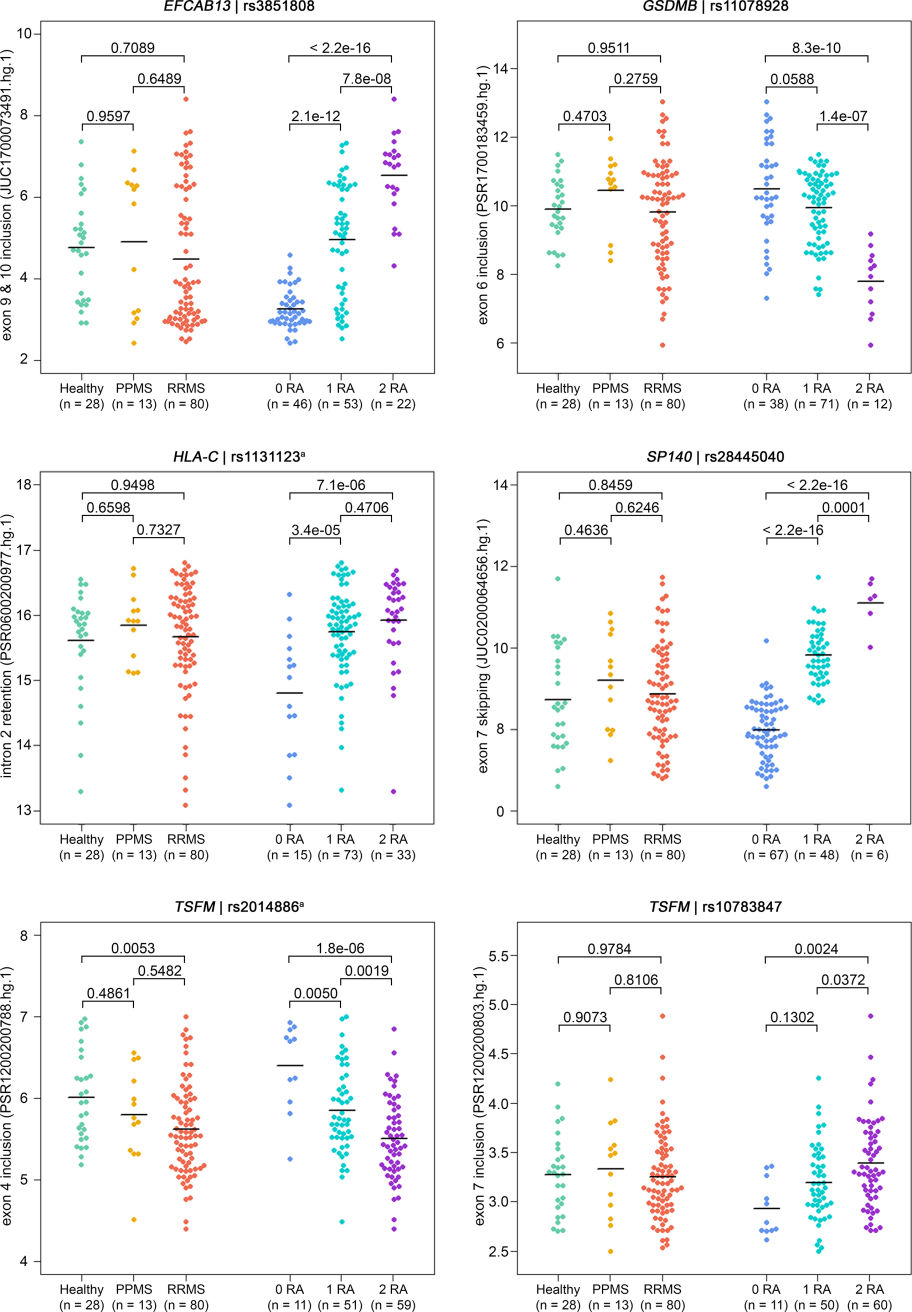
Detection of ASEs in transcriptome data from the B cells of MS patients vs. healthy controls and in relation to splice SNP genotypes. For all 121 samples, the expression of individual exons and exon–exon junctions was interrogated using PSR and JUCs, respectively. Signal intensities (in log2 scale) and group means (black lines) are depicted for the ASEs of the six SNP–gene pairs for which we found significant associations with the genotype (**Table 4**). Shown are the comparisons of expression levels between the three study groups (on the left) and between the splice SNP genotypes (on the right). P-values from pairwise Tukey *post-hoc* analyses and the numbers of samples per group are given. The numbering of exons and introns is as specified in **Table 1**. ^a^ For technical reasons, the designated splice SNP was tagged by a proximal SNP (**Supplementary file**). ASE, alternative splicing event; JUC, junction probe set; MS, multiple sclerosis; PPMS, primary progressive MS; PSR, probe selection region; RA, risk allele; RRMS, relapsing–remitting MS; SNP, single-nucleotide polymorphism.

For six SNP–pairs, the levels of exons and junctions were significantly associated with the genotype of the respective splice SNP (**Figure 2**). In B cells from individuals that were homozygous for the MS risk alleles of the splice SNP, we detected lower levels of *GSDMB* exon 6 and higher levels of the *SP140* exon 6 to exon 8 splice junction. The exons 9 and 10 of *EFCAB13* and the intron 2 of *HLA-C* were found more likely to be included in the mRNA when the MS risk allele is present. Regarding *HLA-C*, we could only evaluate the ASE type intron retention as there are no PSR/JUC probe sets on Clariom D arrays that represent transcripts in which the intron is spliced out. We also found that the two splice SNPs located in the *TSFM* gene are associated with differential alternative splicing. These two SNPs are in the proximity of the same MS SNP, and the respective ASEs presumably account for a short and long transcript isoform of *TSFM* (ENST00000417094 and ENST00000550559). We observed that the levels of the exon 4 of the short transcript were significantly lower in the presence of the MS risk allele C of splice SNP rs2014886, and that the levels of the exon 7 of the long transcript were significantly higher when the MS risk allele G of splice SNP rs10783847 is present. Note that for all six SNP–gene pairs for which the splice SNP genotype was significantly associated with exon- or junction-specific expression levels, the data always correlated with the number of risk alleles carried, i.e., the average expression of the group of heterozygotes was always between that of the two homozygous groups (**Figure 2**). The full results of the transcriptome data analysis, including those for probe sets that capture the respective opposite events, are provided in **Supplementary Tables S9** and **S10** (**Supplementary file**). The transcriptome data are accessible through GEO Series accession number GSE190847.

### Validation of differential splice isoform expression

To confirm that the splice SNPs affect ASEs and consequently the expression of different splice isoforms, we performed qPCR measurements with 109 of the 121 B-cell RNA samples. Based on these data, we compared the expression of mRNA splice isoforms between MS patients and healthy controls and between the splice SNP genotypes (**Table 5**).

**Table 5:** Differential expression of transcript isoforms in the qPCR data set.

Gene (ASE)	MS patients vs. healthy controls				Splice SNP	Genotypes				
	Group (n)	MV	Mean ± SD	p-value		RA (n)	MV	Mean ± SD	p-value
*CLEC16A* (long exon 11)	Healthy (n = 25)	0	75.54 ± 20.26	0.7001	rs11074944	2 RA (n = 100)	0	74.24 ± 26.50	0.0206
	PPMS (n = 11)	0	82.60 ± 20.81			1 RA (n = 9)	0	95.93 ± 26.86	
	RRMS (n = 73)	0	75.20 ± 29.92			0 RA (n = 0)	—	—	
*CLEC16A* (exon 22)	Healthy (n = 25)	0	132.90 ± 52.02	0.0125	rs3214361	genotyping failed			
	PPMS (n = 11)	0	148.62 ± 60.19						
	RRMS (n = 73)	0	108.27 ± 47.59						
*EFCAB13* (exon 9 & 10 inclusion)	Healthy (n = 25)	5	19.77 ± 20.13	0.9909	rs3851808	2 RA (n = 18)	0	59.34 ± 42.59	**3.4e-14**
	PPMS (n = 11)	2	19.41 ± 27.52			1 RA (n = 49)	1	21.29 ± 20.96	
	RRMS (n = 73)	11	20.45 ± 32.60			0 RA (n = 42)	17	2.13 ± 3.74	
*GSDMB* (exon 6 inclusion)	Healthy (n = 25)	0	49.26 ± 50.89	0.8156	rs11078928	2 RA (n = 12)	0	1.19 ± 2.38	**5.4e-06**
	PPMS (n = 11)	0	61.04 ± 51.63			1 RA (n = 64)	0	50.28 ± 37.73	
	RRMS (n = 73)	0	53.02 ± 50.91			0 RA (n = 33)	0	76.99 ± 64.97	
*HLA-C* (without intron 2)	Healthy (n = 25)	0	5673.15 ± 4759.72	0.5373	rs1131123*	2 RA (n = 29)	0	4851.27 ± 3601.61	0.8859
	PPMS (n = 11)	0	4423.68 ± 3353.48			1 RA (n = 65)	0	4989.49 ± 3830.44	
	RRMS (n = 73)	0	4786.48 ± 3489.30			0 RA (n = 15)	0	4993.27 ± 4210.72	
*IL7R* (exon 6 inclusion)	Healthy (n = 25)	0	62.06 ± 35.36	**0.0015**	rs6897932	2 RA (n = 64)	0	40.71 ± 38.50	0.8838
	PPMS (n = 11)	0	17.38 ± 12.75			1 RA (n = 37)	0	46.77 ± 38.83	
	RRMS (n = 73)	0	39.26 ± 37.44			0 RA (n = 8)	0	34.17 ± 10.20	
*NCAPH2* (without intron 19)	Healthy (n = 25)	0	149.13 ± 42.44	0.0495	rs2782	2 RA (n = 18)	0	147.47 ± 54.08	0.7155
	PPMS (n = 11)	0	153.54 ± 43.05			1 RA (n = 60)	0	124.46 ± 46.71	
	RRMS (n = 73)	0	126.43 ± 49.97			0 RA (n = 31)	0	145.94 ± 46.21	
*SP140* (exon 7 skipping)	Healthy (n = 25)	0	88.46 ± 84.25	0.0996	rs28445040	2 RA (n = 5)	0	272.56 ± 129.84	**3.2e-18**
	PPMS (n = 11)	0	153.27 ± 173.82			1 RA (n = 41)	0	156.19 ± 94.40	
	RRMS (n = 73)	0	89.07 ± 78.93			0 RA (n = 63)	0	41.80 ± 28.00	
*TSFM* (exon 3 & 4 inclusion)	Healthy (n = 25)	19	0.12 ± 0.24	0.7139	rs2014886*	2 RA (n = 53)	47	0.02 ± 0.08	**1.2e-05**
	PPMS (n = 11)	9	0.10 ± 0.20			1 RA (n = 45)	24	0.24 ± 0.37	
	RRMS (n = 73)	48	0.16 ± 0.33			0 RA (n = 11)	5	0.35 ± 0.44	
*TSFM* (exon 6 & 7 inclusion)	Healthy (n = 25)	6	1.02 ± 0.95	0.8438	rs10783847	2 RA (n = 54)	9	1.45 ± 2.45	0.1827
	PPMS (n = 11)	3	1.32 ± 0.58			1 RA (n = 44)	6	0.98 ± 0.65	
	RRMS (n = 73)	9	1.25 ± 2.12			0 RA (n = 11)	3	0.90 ± 0.76	

Verification of ASE-dependent transcript expression in B cells by isoform-specific assays in a subset of 109 samples. Shown are group means and standard deviations of the qPCR data that were normalized and transformed to linear scale (Mean ± SD). The number of samples in which the corresponding transcript could not be detected and for which C_T_ values were thus imputed is indicated (MV). The structure of the table is otherwise similar to Table 4, except that for HLA-C the alternative event was considered rather than intron 2 retention due to invalid data for one of the assays used. The full summary statistics for the qPCR data analysis are given in **Supplementary Tables S11 and S12 (Supplementary file)**. Significant expression differences (p < 0.01) are shown in bold. For EFCAB13, GSDMB, SP140 and TSFM, we verified the corresponding ASEs as genotype-dependent. ^*^ For technical reasons, the designated splice SNP was tagged by a proximal SNP (**Supplementary file**). —, not available; ASE, alternative splicing event; MS, multiple sclerosis; MV, missing values; n, number; PPMS; primary progressive MS; RA, risk allele; RRMS, relapsing-remitting MS; SNP, single-nucleotide polymorphism.

**Figure 3:**
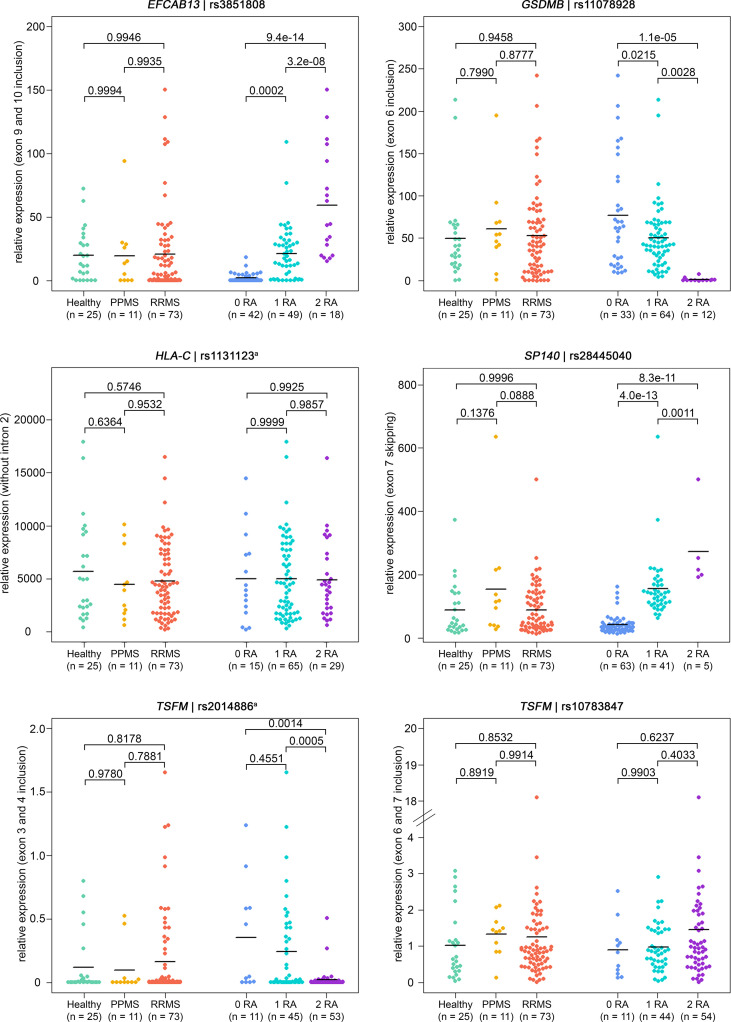
Verification of ASEs in MS patients *vs*. healthy controls and in relation to splice SNP genotypes. Relative expression as measured in B cells by qPCR (n = 109 samples). The same ASEs as in **Figure 2** are visualized (but for *HLA-C* related to the isoform with intron 2 spliced out). Means per group are shown as horizontal black lines. Shown are the comparisons of mRNA isoform expression levels between the three study groups (on the left) and between the splice SNP genotypes (on the right). P-values from pairwise Tukey *post-hoc* analyses and the number of samples for each group are given. The numbering of exons and introns is as specified in **Table 1**. ^a^ For technical reasons, the designated splice SNP was tagged by a proximal SNP (**Supplementary file**). ASE, alternative splicing event; MS, multiple sclerosis; PPMS, primary progressive MS; qPCR, quantitative real-time PCR; RA, risk allele; RRMS, relapsing-remitting MS; SNP, single-nucleotide polymorphism.

Overall, the qPCR data well reflected the transcriptome data. In line with the transcriptome data, we saw significantly higher levels of *IL7R* transcripts that contain exon 6 in the qPCR data of healthy controls compared to those of MS patients. In addition, in the presence of the MS risk allele, exons 9 and 10 of *EFCAB13* were included more frequently, exon 7 of *SP140* was skipped more frequently and exon 6 of *GSDMB* and exons 3 and 4 of *TSFM* were included at significantly lower rates (**Figure 3**). In the case of *TSFM* | rs2014886, the short transcript isoform (ENST00000417094) is only rarely expressed in B cells, which explains the high number of missing values. For splice SNP rs10783847 and *TSFM* exons 6 and 7 (ENST00000550559), a non-significant trend toward preferential exon inclusion has been observed for the carriers of the MS risk allele. In contrast to the transcriptome data, no genotype dependence of the ASE in *HLA-C* (intron 2 retention) was seen in the qPCR data. The detailed results of the qPCR analysis are available in **Supplementary Tables S11** and **S12** (**Supplementary file**).

### Multiple sclerosis–associated splice single-nucleotide polymorphism affects splicing pattern of *TSFM*

Since evidence of genotype-dependent splicing was found for 6 of the 10 SNP–gene pairs within the transcriptome and/or qPCR data for our study cohort, we used splicing reporter minigene assays to investigate whether the ASEs are causally related to the splice SNP allele variants. We focused on the seven ASEs that have not yet been previously studied in the samples of MS patients according to our recent systematic review (49), i.e., for *CLEC16A*, the alternative 5’ donor site and the alternative last exon, exon skipping for *EFCAB13* and *TSFM,* and intron retention for *HLA-C* and *NCAPH2* (**Table 1**).

**Figure 4:**
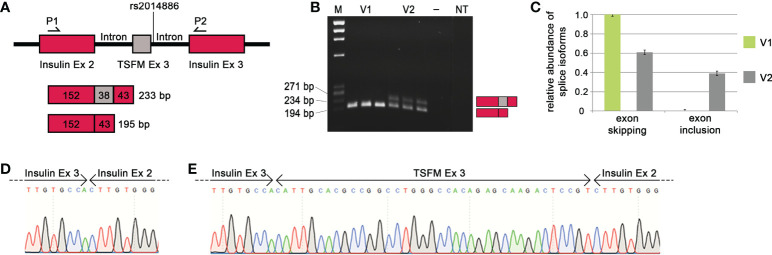
Effect of MS-associated splice SNP on *TSFM* exon 3 skipping. **(A)** Simplified depiction of the minigene assay for *TSFM*. The sequences of *TSFM* exon 3 (gray box) as well as 400 bp of the up- and downstream introns were cloned between rat insulin exon 2 and 3 (burgundy boxes) of the pDESTsplice vector. The splice SNP rs2014886 is located in the intronic region 2 nucleotides downstream of exon 3. The matching sequences for the PCR primers are located in the sequences of the rat insulin exons (P1 and P2). If *TSFM* exon 3 is included in the resulting transcript, the PCR product has a size of 233 bp. If exon 3 is skipped, the resulting PCR product has a size of 195 bp. **(B)** The PCR products for the variants V1 and V2 (from triplicate measurements) were visualized by gel electrophoresis. V1 represents the MS risk allele C and V2 represents the alternative allele T of splice SNP rs2014886. **(C)** The relative proportions of splice isoforms that resulted due to *TSFM* exon 3 skipping or *TSFM* exon 3 inclusion for the allele variants V1 (green) and V2 (gray). The MS risk allele C of splice SNP rs2014886 favors *TSFM* exon 3 skipping. The two splice isoforms were verified by reverse direction sequencing **(D, E)**. —, negative control; bp, base pairs; Ex, exon; M, size standard; MS, multiple sclerosis; NT, non-template control; P1, PCR_RatInsEx2; P2, PCR_RatInsEx3 (**Supplementary Table S6**, **Supplementary file**); SNP, single-nucleotide polymorphism.

When the MS risk allele of the splice SNP rs2014886 is present, there is a C two nucleotides downstream of *TSFM* exon 3 (variant V1). In this case, we observed *TSFM* exon 3 skipping (**Figure 4**). On the other hand, when the minigene construct carried the alternative allele T (variant V2), exon 3 was frequently included between the constitutively expressed rat insulin exons (**Figures 4B, C**). More precisely, the creation of the donor splice site due to the allele T resulted in a significant shift in the expression of the transcript isoforms: from a proportion of nearly 100% exon skipping to a proportion of 61% exon skipping and 39% exon inclusion (*p* = 2.8e-09). We verified that the ASE of *TSFM* depends on splice SNP rs2014886 by sequencing (**Figure 4D**). These findings are in line with the results from the analyses of B cells with microarrays and qPCR assays (**Tables 4**, **5**).

We also observed a preferential intron 2 retention for *HLA-C* related to the MS risk allele T of SNP rs1131123 (**Figure S3**). In the presence of the allele T, we saw a shift of the relative proportion of intron 2 retention from 67% to 87% (*p* = 3.6e-08). This is consistent with the observations from the microarray data analysis (**Figure 2**). However, for the other five SNP–gene pairs (*CLEC16A* | rs11074944, *CLEC16A* | rs3214361, *EFCAB13* | rs3851808, *NCAPH2* | rs2782 and *TSFM* | rs10783847), similar relative proportions of the different transcription products were obtained independently of the allelic variant, and the tests for interactions did not reach the significance level. Thus, we could not confirm that these ASEs are causally related to the splice SNP genotypes in the minigene assays (**Figure S3**).

## Discussion

In this study, we combined *in silico* evaluations to identify SNPs that may alter pre-mRNA splicing with expression analyses of B cells and with cell culture experiments. We demonstrate that the genotype of SNPs in LD with MS-associated genetic variants can affect pre-mRNA splicing and thus the expression of splice isoforms. We observed an association of the splice SNP genotype with the expression of exons and exon–exon junctions for six SNP–gene pairs (*EFCAB13* | rs3851808, *GSDMB* | rs11078928, *HLA-C* | rs1131123, *SP140* | rs28445040, *TSFM* | rs10783847, and *TSFM* | rs2014886) in the microarray data. The differential alternative splicing could be verified by qPCR analyses for *EFCAB13*, *GSDMB*, *SP140*, and *TSFM*. With our findings for *GSDMB* and *SP140*, we could support previous results in the literature showing that the MS-associated SNPs affect alternative splicing (49).

As a starting point, we used various bioinformatic tools to prioritize genetic variants that are likely to alter the pre-mRNA splicing of MS risk genes. We here focused on SNPs located in an exon or within 400 bp of the adjacent intronic regions of these genes. According to previous studies, most splicing factor motifs can be found within this selected 400 bp window (59–61). For the prediction of splicing events due to genetic variants, different tools and databases are available (62–64). We used the Human Splicing Finder to investigate whether a SNP may affect a *cis*-element such as a branch point, a splice site, or an exonic/intronic splicing enhancer or silencer, and we used the POSTAR2 database to identify SNPs in experimentally determined RBP-binding sites. Finally, we determined 10 SNP–gene pairs (10 SNPs and 8 different genes) for the further event-focused investigations. The reliability of our splice SNP selection procedure was supported by the identification of ASEs for *GSDMB* | rs11078928, *IL7R* | rs6897932, and *SP140* | rs28445040 as an aberrant genotype-dependent splicing in MS has been previously described for these three SNP–gene pairs (49), and the SNPs are also reported as sQTL SNPs for whole-blood and EBV-transformed lymphocyte samples in the GTEx portal (34). Most of the eight prioritized genes are expressed with a low immune cell type specificity according to the Human Protein Atlas (65). However, two of the genes are expressed more specifically in certain immune cell types: *IL7R* is expressed mainly in the subsets of T cells and natural killer cells, and *SP140* is expressed mainly in memory B cells (66).

Then, we examined the association between the genotype of splice SNPs with the expression of the genes as well as with the levels of individual exons/junctions of the distinct splice isoforms of these genes in B cells from MS patients and healthy controls. Apart from the fact that we did not include SPMS patients, the group of MS patients resembled the typical characteristics of MS patients in European MS registries in terms of age, disease status, and sex (67). In line with the literature (50, 68–70), we observed a significant differential expression of exon 6 of *IL7R* in MS patients as compared to healthy controls. In our data, the levels of transcripts containing exon 6 were lower in MS patients, but we could not find the previously described association to the MS risk allele C of the non-synonymous splice SNP rs6897932 (T244I). However, the latter might result from the fact that we studied the expression in B cells and not in T cells, in which *IL7R* is more strongly expressed (66). *IL7R* encodes for a cell surface receptor for interleukin-7, which plays an essential role for the development and survival of T cells (71). Gregory et al. reported that the C allele of SNP rs6897932 augments an exonic splicing silencer and thus promotes exon 6 skipping, leading to a splice isoform that encodes a soluble form of the protein (50). This is of relevance as increased levels of soluble interleukin-7 receptor have been shown to exacerbate the disease severity in an EAE mouse model, presumably by increasing the activity or bioavailability of interleukin-7 (72). Our analyses of B-cell RNA samples by microarrays and qPCR indicated a genotype-dependent skipping of *GSDMB* exon 6 and *SP140* exon 7. Consistent with our findings, Cardamone et al. (68), Garrido-Martín et al. (73), and Morrison et al. (74) found that the MS risk allele C of SNP rs11078928 affects the acceptor splice site of *GSDMB* exon 6, resulting in increased exon 6 skipping. The encoded protein Gasdermin-B mediates pyroptosis (75) and, in addition to MS, genetic variants in the *GSDMB* gene have also been associated with susceptibility to other multifactorial autoimmune diseases like rheumatoid arthritis (76) and ulcerative colitis (77). With regard to the genotype-dependent splicing of *SP140*, Cardamone et al. (78) and Matesanz et al. (79) could demonstrate *via* minigene assays that the MS risk allele T of SNP rs28445040 leads to the skipping of exon 7. The function of the protein encoded by *SP140* is only partially known. However, the presence of chromatin-related protein domains indicates a role in the chromatin-mediated regulation of gene expression (80). In addition, Karaky et al. reported that SP140 regulates the expression of immune-related genes that are associated with MS (81).

For four other SNP-gene pairs (*EFCAB13* | rs3851808, *HLA-C* | rs1131123, *TSFM* | rs2014886, and *TSFM* | rs10783847), we could detect differential alternative splicing in B cells in relation to the MS risk allele. We observed increased *EFCAB13* expression and preferential inclusion of exons 9 and 10 in the presence of the MS risk allele C of splice SNP rs3851808. The genotype dependency of this ASE is supported by an sQTL association that is reported for *EFCAB13* | rs3851808 for EBV-transformed lymphocytes and other cell types and tissues in the GTEx portal (34). The protein encoded by *EFCAB13* contains a calcium-binding domain that is shared by a variety of calcium sensor proteins, which play a role in neuronal function and plasticity (82, 83). Diseases implicated with calcium sensor proteins are, for instance, Alzheimer’s disease (84) and various cancer types (85, 86).

For *HLA-C* | rs1131123, we observed a trend toward preferential *HLA-C* intron 2 retention in the presence of the MS risk allele T of the non-synonymous splice SNP rs1131123 (D114A). This genotype dependency was also observed with the minigene assay. *HLA-C* encodes a class I major histocompatibility complex antigen. Class I molecules play a central role in the immune system and have repeatedly been demonstrated to contribute to the genetic susceptibility to MS (87–89). However, there were challenges in examining the ASE of pre-mRNA from *HLA-C*: first, in the microarray data, we could only evaluate the expression of the transcript variant in which intron 2 is retained in the mRNA because there are no PSR/JUC probe sets for *HLA-C* intron 2 exclusion on the employed chip model. Second, only one of the two qPCR assays used to measure transcript splice isoforms of *HLA-C* provided valid data, which might be due to a sensitivity of the primer pair toward *HLA-C* subtypes. Since the SNP rs1131123 is not recorded in the GTEx portal (34), further investigations, e.g., with RNA sequencing, could be helpful to ascertain the presumed genotype-dependent splicing of *HLA-C* intron 2.

We found that exons 3 and 4 of the short transcript variant ENST00000417094 are more frequently skipped in the presence of the MS risk allele C of splice SNP rs2014886 and that this short transcript is only rarely expressed in B cells. In line with our B-cell transcriptome data and minigene assay data, an association of the C allele of SNP rs2014886 with *TSFM* exon 3 skipping was previously reported by Morrison et al. (74). However, they only studied a small study cohort of eight individuals per genotype. In a recently published report, which focused exclusively on the identification of potential cryptic exons based on literature reports and the dbSNP database, a genotype-dependent splicing of *TSFM* exon 3 was also postulated (90). Moreover, an sQTL that links the skipping of *TSFM* exon 3 and 4 with SNP rs2014886 is listed for EBV-transformed lymphocytes in the GTEx portal (34). For the second SNP–gene pair with *TSFM*, we found that the MS risk allele G of splice SNP rs10783847 showed a strong trend toward *TSFM* exon 6 and 7 inclusion of the transcript isoform ENST00000550559. *TSFM* encodes for a mitochondrial translation elongation factor, which catalyzes the exchange of GDP to GTP (91, 92). As the respiratory chain function relies on proper mitochondrial gene expression, differential *TSFM* expression is associated with various diseases such as encephalomyopathy, hypertrophic cardiomyopathy, and MS (93–96). Noteworthy, Alcina et al. (96) described that SNP rs10877013 affects *TSFM* expression in MS by altering the enhancer activity of a regulatory element. This SNP is in almost perfect LD with the two splice SNPs rs10783847 and rs2014886 in the European population (33). Further studies are needed to better understand the functional role of the different splice isoforms of *TSFM* in relation to the pathogenesis of the multifactorial disease MS. According to the Ensembl database, ENST00000417094 codes for an 89 amino acid long protein sequence (UniProt F8WCK2) but it is likely a target of nonsense-mediated decay. However, experimental evidence remains to be established.

The following limitations should be considered when interpreting the data of this study. First, due to the stringent restrictions on the selection of experimentally screenable SNP–gene pairs, it is possible that we have missed some MS-specific ASEs. For instance, we did not include rare variants (minor allele frequency < 1%) because the sample size would be insufficient to study such variants. In addition, we focused only on SNPs in or near exons and thus did not capture the potential influence of deep intronic SNPs on splicing. Such variants have been described for other diseases (61, 97–100). Second, some genomic regions are characterized by long-range LD. Hence, the observed effects on splicing may not represent the only effects underlying the genetic associations with MS. Third, in the analyses of differences in gene expression and alternative splicing, we cannot exclude the possibility of confounding variables, e.g., medical treatment and comorbidities. Specifically, we observed a shift in the proportions of B-cell subsets in patients treated with alemtuzumab or cladribine (53). This contributed to the variance in the gene expression data. Fourth, we conducted our measurements in B cells and therefore may have missed or underestimated the differential alternative splicing of genes that are more abundantly expressed in other cell types (101). Even though genetic effects on splicing are usually highly shared across tissues and cell types (34), further insights into the effects of genetic risk variants could be obtained by studying other cell types, e.g., other peripheral immune cells such as T cells. Fifth, our analysis of the microarray data relied on transcript isoforms as annotated in the reference genome. Thus, we studied known splice isoforms and may have missed novel splicing patterns, which can potentially be identified by using RNA sequencing (102, 103). Fifth, as we have previously described (46), there are issues regarding the use of the minigene assay system, such as a possible interference by the Gateway cloning attachment sites, an insufficient amount of an important splicing factor in the used cell line, or the fact that only a small and specific part of the gene is examined. The latter may lead to the misinterpretation of ASEs as the splicing of exons can depend on the correct splicing of other exons of the gene that are not included in the minigene construct.

In conclusion, in this study, we focused on SNPs located in genetic risk loci for MS that presumably affect pre-mRNA splicing and thus may have an influential role in the pathogenesis of the disease. We were able to support findings from previous studies on MS-related ASEs for the pre-mRNAs of *GSDMB*, *IL7R*, and *SP140*. For four novel SNP–gene pairs, we found an association of the splice SNP genotypes with differential alternative splicing in the B-cell transcriptome data. Except for two SNP–gene pairs, we were able to validate the findings of the microarray data analysis with the qPCR assays. In addition, we were able to further substantiate our observations from the B-cell expression data on *TSFM* exon 3 skipping by using minigene assays. The MS risk allele C of the SNP rs2014886 almost always led to *TSFM* exon 3 skipping, whereas the alternative allele led to a low expression of ENST00000417094 transcripts. However, the potential functional impact of this ASE remains unclear. Further functional studies are needed to identify the disease-causing genetic variants and to explore their effects on splicing and the resulting consequences of an aberrant expression of splice isoforms to improve our understanding of the molecular pathomechanisms of MS.

## Data availability statement

The datasets presented in this study can be found in online repositories. The names of the repository/repositories and accession number(s) can be found in the article/**Supplementary Material**.

## Ethics statement

The studies involving human participants were reviewed and approved by the Ethics committee of the University of Rostock. The patients/participants provided their written informed consent to participate in this study.

## Author contributions

EP performed the bioinformatic prioritization of SNP-gene pairs as well as the majority of the experiments. EP evaluated and interpreted the obtained data. Furthermore, EP drafted the manuscript and prepared the figures and tables. MH provided support for the bioinformatic and statistical analyses of the data and critically revised the manuscript. MS coordinated the sample collection and gathered the clinical-demographic data. EP, NB and BF processed the blood samples. NB performed the SNP genotyping. DK was responsible for performing the microarray measurements. PL provided valuable advice concerning the splicing reporter minigene assays. MH, BF and UKZ conceptualized the study. MH, EP, NB and UKZ secured research funding. UKZ provided important intellectual insights and supervised the research. All authors contributed to the article and approved the submitted version.

## Funding

EP received a scholarship from the Landesgraduiertenförderung Mecklenburg-Vorpommern. NB was funded by the Stiftung der Deutschen Wirtschaft (sdw). Research funding for this work was provided by the Rostock University Medical Center (FORUN program, grant 889034). In addition, this study was partially funded by Sanofi Genzyme (grant GZ-2016-11560) and Merck Serono GmbH (Darmstadt, Germany, an affiliate of Merck KGaA, CrossRef Funder ID: 10.13039/100009945, grant 4501860307). The funder was not involved in the study design, collection, analysis, interpretation of data, the writing of this article or the decision to submit it for publication.

## Acknowledgments

We thank Alexander Winkelmann, Stefanie Meister, and Ales Dudesek for patient care and Antje Bombor and Ina Schröder for coordinating the clinical visits and blood draws. We wish to thank Nele Retzlaff and Deborah Sonnenberg for reviewing the medical records. Our thanks go to Hans-Jürgen Thiesen, and Robert Jaster for providing access to technical equipment. We thank the Core Facility for Cell Sorting and Cell Analysis headed by Brigitte Müller-Hilke for providing the FACS equipment and technical support. We are grateful to Ildikó Tóth for laboratory support and to Felix Steinbeck for his input on computational tools for analyzing genetic data.

## Conflict of interest

EP received travel funds from Novartis. MH received speaking fees and travel funds from Bayer HealthCare, Biogen, Merck, Novartis, and Teva. UKZ received research support as well as speaking fees and travel funds from Alexion, Almirall, Bayer HealthCare, Biogen, Bristol Myers Squibb, Janssen, Merck Serono, Novartis, Roche, Sanofi Genzyme, and Teva as well as EU, BMBF, BMWi, and DFG.

The remaining authors declare that the research was conducted in the absence of any commercial or financial relationships that could be construed as a potential conflict of interest.

## Publisher’s note

All claims expressed in this article are solely those of the authors and do not necessarily represent those of their affiliated organizations, or those of the publisher, the editors and the reviewers. Any product that may be evaluated in this article, or claim that may be made by its manufacturer, is not guaranteed or endorsed by the publisher.

## Glossary

**Table:**

—	not available
Alt.	alternative
ANOVA	analysis of variance
ASE	alternative splicing event
bp	base pairs
CNS	central nervous system
C_T_	threshold cycle
dist.	distance
EAE	experimental autoimmune encephalomyelitis
EBV	Epstein–Barr virus
EDSS	Expanded Disability Status Scale
EDTA	ethylenediaminetetraacetic acid
eQTL	expression quantitative trait locus
ESE	exonic splicing enhancer
ESS	exonic splicing silencer
EUR	European population
GEO	Gene Expression Omnibus
GWAS	genome-wide association study
h	hours
HGNC	HUGO Gene Nomenclature Committee
ISE	intronic splicing enhancer
ISS	intronic splicing silencer
JUC	junction probe set
kb	kilobase
LD	linkage disequilibrium
mRNA	messenger RNA
MS	multiple sclerosis
MS RA	MS risk allele
MS SNP	MS-associated lead SNP according to the most recent GWAS (32)
MV	missing values
n	number
ng	nanogram
PBMC	peripheral blood mononuclear cells
PPMS	primary progressive multiple sclerosis
pre-mRNA	precursor messenger ribonucleic acid
PSR	probe selection region
qPCR	quantitative (real-time) polymerase chain reaction
RA	risk allele
RBP	RNA-binding protein
RRMS	relapsing–remitting multiple sclerosis
RT	reverse transcription
RT-PCR	reverse transcription polymerase chain reaction
SD	standard deviation
SNP	single-nucleotide polymorphism
snRNPs	small nuclear ribonucleoproteins
SNP splice	potentially splice-altering SNP that is located in or near an exonic region of a gene and that is in LD with an MS SNP
SPMS	secondary progressive multiple sclerosis
sQTL	splicing quantitative trait locus
TAC	Transcriptome Analysis Console
