# Supplementary material for: Genetic risk variants for multiple sclerosis are linked to differences in alternative pre-mRNA splicing
Source: Front Immunol. 2022 Oct 28;13:931831. doi: 10.3389/fimmu.2022.931831 (PMC9670805; doi:10.3389/fimmu.2022.931831)
Supplement: Supplementary file 1 [file DataSheet_1.pdf]

Supplementary Material for

Genetic risk variants for multiple sclerosis are linked to differences in alternative pre-mRNA splicing

Elena Putscher[a], Michael Hecker[a,*], Brit Fitzner[a], Nina Boxberger[a], Margit Schwartz[a], Dirk Koczan[b], Peter Lorenz[b], Uwe Klaus Zettl[a]

[a] Rostock University Medical Center, Department of Neurology, Division of Neuroimmunology, Gehlsheimer Str. 20, 18147 Rostock, Germany

[b] Rostock University Medical Center, Institute of Immunology, Schillingallee 70, 18057 Rostock, Germany

* corresponding author: Dr. Michael Hecker, e-mail: michael.hecker@rocketmail.com, phone: +49 381 494-5890, fax: +49 381 494-5882

ORCID: 0000-0001-7015-3094

Short title: MS-associated SNPs influence alternative pre-mRNA splicing

MATERIAL AND METHODS

Unless otherwise stated, all materials were used according to the manufacturers' instructions.

Bioinformatic prioritization and characterization of SNP-gene pairs

Using publicly available Affymetrix Human Transcriptome Array (HTA) 2.0 data sets (Table S1), we compared the transcriptome of blood cells from healthy subjects with that of five cell populations from the peripheral blood of MS patients. HTA 2.0 microarrays contain over 6 million different probes (25 base long oligonucleotides). An exonic region is typically split into different exon parts or fragments to consider the overlap among transcript isoforms [1]. For each exon fragment (probe selection region, PSR), there are 10 probes. For each exon-exon junction (JUC) that corresponds to annotated transcript isoforms, there are 4 probes. This microarray design allows deep insights into alternative splicing patterns. For the primary data analysis, the default settings (e.g., SST-RMA normalization) of the Transcriptome Analysis Console (TAC, version 4.0.2) software were used. The SST-RMA (Signal Space Transformation Robust Multi-Array Average) method was applied for background adjustment, quantile normalization, probe set summarization and log2 transformation. To identify differentially spliced genes, we required a splicing index > 4 or < -4 and an EventPointer [2] p-value < 1.0e-07 in all 20 comparisons of data sets for MS patients with data sets for controls. Moreover, we included all genes for which alternative splicing in MS has been previously studied in the literature according to our systematic review [3]. We then focused on the genes that are located within a distance of up to 250 kb of an MS-associated lead SNP from the GWAS [4] (MS SNP). By using the webtool LDproxy [5], we determined SNPs that are in linkage disequilibrium (LD) with an MS SNP ($r^2 > 0.1$ and $D' > 0.7$) and that are located within the genes. We narrowed down the selection of SNPs to those located within exons or the adjacent intronic regions (up to 400 bp from the exon). Subsequently, by using the splicing prediction tool HSF 3.1 [6] and information from the POSTAR2 database [7] on splicing-related RNA-binding proteins (RBPs) [8–12], we searched for SNPs that potentially

regulate pre-mRNA splicing (splice SNP). Then, SNP-gene pairs were selected for experimental validation by the following four points:

1) The splice SNP alters a wild-type donor splice site, acceptor splice site or branch point sequence

2) According to the TAC analysis, an exon / intron is processed differently in MS than in healthy subjects and either in HSF 3.1 or in POSTAR2 it is indicated that the splice SNP alters an exonic splicing silencer or enhancer (ESS/ESE) or an RBP binding site, respectively

3) The gene has already been studied in the literature with respect to aberrant splicing in MS [3] and either in HSF 3.1 or in POSTAR2 it is indicated that the splice SNP alters an ESS/ESE or an RBP binding site, respectively

4) For the splice SNP, both HSF 3.1 and POSTAR2 indicate a change in an ESS/ESE motif and an RBP binding site, respectively

In the final step, we assessed whether the prioritized splice SNPs are related to ASEs that distinguish at least two protein-coding transcript isoforms according to the Ensembl database (release 93).

Blood sample collection and DNA extraction

Twenty ml of peripheral blood were collected from MS patients and healthy controls by venipuncture into tubes with ethylenediaminetetraacetic acid (EDTA) as anticoagulant. In comparison to our former study [13], we included one more sample from one of the RRMS patients receiving alemtuzumab in the present study. DNA was extracted from 200 µl of whole blood using the QIAamp DNA blood mini kit (Qiagen) and stored at -20 °C.

SNP genotyping

PCR-based genotyping was performed with custom TaqMan® Array Cards (Thermo Fisher Scientific) (Table S2). In each of the wells, sequence-specific forward and reverse primers and two allele-specific TaqMan® minor groove binder probes labeled with either VIC® or

FAM™ were preplated. For two splice SNPs, due to technical constraints, SNPs in high or perfect LD with the splice SNP were selected: rs2014886 was tagged by rs1599932 and rs1131123 was tagged by rs41543814. For each reaction, ~4 ng of DNA diluted in TaqMan® Universal Master Mix II with UNG (Applied Biosystems) was utilized. PCR amplification was performed in 40 cycles with a ViiA 7 Real-Time PCR System (Applied Biosystems). The raw data were analyzed in an automated manner using the TaqMan Genotyper Software (version 1.6). After manual validation of the allelic discrimination, the SNP rs3214361 was excluded from further analyses (genotyping failed).

B-cell isolation

From the whole blood samples, peripheral blood mononuclear cells (PBMC) were isolated using Ficoll density gradient separation (Histopaque-1077, Sigma-Aldrich). B cells were then isolated from the PBMC via a negative selection approach using the Pan B cell isolation kit for human (Miltenyi Biotec). The cells were sorted using an autoMACS Pro magnetic separator (Miltenyi Biotec). After separation, the negative fraction contained mainly B cells. The number of B cells was determined by using a Neubauer counting chamber. The purity of the B cells was checked by flow cytometry using a FACSCalibur instrument (BD Inc.) and different antibodies (Table S3) as described previously [13].

B-cell RNA extraction

The B cells were lysed in QIAzol lysis reagent (Qiagen miRNeasy Mini Kit), and the lysates were stored at -80 °C until further use. Upon completion of the blood sample collection, total RNA was extracted using the miRNeasy Mini Kit and the RNase-free DNase Set (Qiagen). The RNA integrity was checked by using RNA 6000 Pico kits and a 2100 Bioanalyzer (Agilent Technologies). The RNA concentrations were measured with a NanoDrop® ND-1000 spectrophotometer (Thermo Fisher Scientific). The purified B-cell RNA samples were stored at -20 °C.

Transcriptome analysis

The B-cell RNA profiling was performed with high-resolution Clariom D microarrays for human (Applied Biosystems). This means that along with measuring the expression of more than 130,000 protein-coding and non-protein-coding genes (transcript clusters, TC probe sets) for gene-level studies (differential gene expression), PSR / JUC probe sets (with six probes and four probes per probe set, respectively) can be evaluated for exon-level studies (differential alternative splicing).

The sample preparation for the microarray measurements was performed with 100 ng of total RNA per sample and the GeneChip WT PLUS Reagent Kit (Applied Biosystems). Hybridization of the obtained amplified, fragmented and biotinylated single-stranded sense strand DNA onto the Clariom D arrays was performed in a GeneChip Hybridization Oven 645 (Affymetrix). Subsequently, the microarrays were washed and stained in a GeneChip Fluidics Station 450 (Affymetrix) and then scanned with a GeneChip Scanner 3000 7G (Affymetrix). To extract the signal intensities for the > 6.7 million 25mer oligonucleotide probes per array, the scans were imported into the Affymetrix GeneChip Command Console version 4.0.

We analyzed the preprocessed microarray data with the TAC software (version 4.0.2) using the default settings. For quality control, we checked the signal intensities of the hybridization controls and of the positive and negative controls as well as the distributions of the raw signal intensities. With a principal component analysis, we inspected the data for outliers (Figure S2). Tukey biweight robust means, standard deviations and p-values were calculated with the TAC software. The statistical testing was based on the limma method with eBayes correction [14]. Only the data of TC / PCR / JUC probe sets concerning the prioritized ten SNP-gene pairs and the respective ASEs were considered in our analyses.

Validation of differential transcript isoform expression via qPCR

The analysis of transcript isoform expression was performed with custom TaqMan® Gene Expression Array Cards (Thermo Fisher Scientific). To measure specific transcript isoforms,

a total of 19 assays were chosen for the 10 SNP-gene pairs (Table S4). Reverse transcription (RT) was performed with 200 ng RNA per sample and the High-Capacity cDNA Reverse Transcription Kit (Applied Biosystems). The cDNA was diluted with TaqMan Universal Master Mix II with UNG (Applied Biosystems). The qPCR measurements were performed with ~1 ng cDNA per reaction using a ViiA 7 Real-Time PCR system (Applied Biosystems) with 45 amplification cycles. The qPCR data were analyzed in an automated manner using the ExpressionSuite software (version 1.3, Thermo Fisher Scientific) to obtain C_T values. Missing values were imputed using the R package nondetects [15]. For data normalization (calculation of ΔC_T values), the mean of three reference genes (GAPDH: Hs99999905_m1, HPRT1: Hs02800695_m1 and UBC: Hs00824723_m1) was used. The data were then converted to the linear scale ($2^{-\Delta C_T}$×1,000).

Plasmid preparation for the minigene assays

The sequences of the targeted exons and 400 nucleotides of the adjacent intronic sequences of seven SNP-gene pairs were retrieved for the minigene assays via the UCSC Genome Browser [16] (human assembly Dec. 2013 (GRCh38/hg38)). Gene synthesis and cloning into pDONR221 vectors of 14 insert sequences (2 allelic variants per SNP-gene pair) was performed by BioCat GmbH (Table S5). For obtaining both allelic variants of each splice SNP, the company performed site-specific mutageneses.

The generation of the minigene constructs relied on the Gateway cloning system. The LR Clonase Enzyme Mix II (Invitrogen) was used for the LR reaction. Per reaction, 150 ng of pDONR221 vector and 150 ng of pDESTsplice vector were applied. No minigene construct could be generated via the LR reaction for *EFCAB13* exon 9 sequence inserts. Therefore, we performed a classical cloning procedure for these two minigene constructs using the restriction enzymes ApaI, Ecl136II (SacI) and Eco32I (EcoRV) (10 U/µl, Fermentas). A ratio of 1:3 of vector and sequence insert and a T4 DNA ligase (Thermo Fisher Scientific) were used to clone the *EFCAB13* sequences into the pDESTsplice vector.

One Shot™ TOP10 Chemically Competent *E. coli* (Invitrogen) were transformed with the minigene constructs. The bacteria were then plated on LB agar plates (with ampicillin) and incubated overnight at 37 °C. We verified the successful recombination of the minigene constructs with mini lysates that were prepared according to a modified protocol by Birnboim and Doly [17]. Accordingly, bacterial clones were picked from the LB agar plate and cultured overnight in 2 ml TY medium with ampicillin at 37 °C and 200 rpm. Plasmid solution 1 (50 mM Glucose, 10 mM EDTA, 25 mM Tris HCl, pH 6 - 8), plasmid solution 2 (0.2 M NaOH, 1% SDS) and plasmid solution 3 (3 M potassium acetate, 11.5% acetic acid) were used for the alkaline lysis of the bacteria. DNA was precipitated by adding isopropanol and washed with 70% ethanol. The DNA was redissolved in H_2O with RNase (10 ng/µl) and incubated for 15 min with shaking. For restriction digestion with KpnI (10 U/µl, Thermo Fisher Scientific), the samples were incubated for 30 – 60 min at 37 °C. The products of restriction digestion were visualized by gel electrophoresis.

For plasmid amplification, we added 2 – 3 µl of the remaining bacterial suspension to 25 ml LB medium with ampicillin. The bacteria were incubated overnight at 37 °C and 200 rpm. Plasmid isolation was performed using the EndoFree Plasmid Maxi Kit with QIAfilter Midi Cartridges and QIAGEN-tip 100 columns (Qiagen). The isolated DNA was eluted in endotoxin-free H_2O. DNA concentration was measured with a NanoDrop® ND-1000 spectrophotometer (Thermo Fisher Scientific). Successful cloning was confirmed by control sequencing that was performed with 80 ng/µl plasmid DNA at Microsynth Seqlab GmbH using the primers seq_RatInsEx2 and seq_RatInsEx3 (Table S6). The plasmid DNA was stored at -20 °C.

Transfection and RNA isolation of HeLa cells

HeLa cells were cultured in DMEM (5.6 g/L glucose + pyruvate, Gibco) + 10% FCS and 50 U/ml penicillin-streptomycin at 37 °C and 5% CO_2. For maintenance culture, the cells were passaged two times per week. The cells were washed with PBS (Gibco) and detached from

the culture flask with Trypsin-EDTA (Gibco) for passaging or for seeding for transfection experiments.

For transfection, we seeded 75,000 cells/well in 12-well plates. After 72 h, the HeLa cells were transfected with the minigene plasmid DNA in biological triplicates. Transfection was performed using FuGENE® HD (Promega) transfection reagent (3 µl FuGENE/well to 1 µg plasmid DNA) and OPTI-MEM® I medium (Gibco). After adding the transfection mixture, the cells were incubated for 24 h at 37 °C and 5% CO_2. Subsequently, we isolated the RNA with the RNeasy Plus Kit (Qiagen). RNA concentrations were measured via a NanoDrop® ND-1000 spectrophotometer (Thermo Fisher Scientific). The RNA samples were stored at -20 °C.

RT-PCR

For the RT reaction, we used the High-Capacity cDNA Reverse Transcription Kit (Applied Biosystems) to transcribe 1,000 ng RNA from HeLa cells into cDNA. The PCR reaction was carried out with the AmpliTaq Gold™ DNA Polymerase with Buffer I (Applied Biosystems), sequence-specific primers (Table S6) and the GeneAmp® PCR System 9700 (Applied Biosystems) with 35 amplification cycles. Based on the used primers, we chose an annealing temperature of 60 °C. Each PCR run included a negative control (RNA instead of cDNA template) and a non-template control (H_2O instead of cDNA template).

The PCR products were visualized by gel electrophoresis. ΦX174 DNA/BsuRI (HaeIII) Marker (0.5 µg/µl, Thermo Fisher Scientific) was used as the size standard. Images of the gels were used to evaluate the distribution of splice isoforms (bands on the gels). The intensity of the bands minus the background signal was determined with the Image Studio Lite software version 5.2 (LI-COR Biosciences). Means and standard deviations of the data from the triplicates were calculated. Subsequently, the relative proportions of splice isoforms were determined and plotted as bar charts. For statistical tests, we performed two-way ANOVAs considering the interaction of splice SNP allele and splice isoform.

The gel bands were purified from a preparative gel. DNA purification was performed with the NucleoSpin Gel & PCR Clean-Up Kit (Macherey-Nagel). DNA concentrations were measured with a NanoDrop® ND-1000 spectrophotometer (Thermo Fisher Scientific). The purified PCR products were cloned into pGEM®-T vectors (Promega). Per reaction, 20-60 ng/µl of the PCR product was applied. One Shot™ TOP10 Chemically Competent *E. coli* (Invitrogen) were used for transformation. Plasmid isolation was performed as described in section "Plasmid isolation". Depending on the sequences, Cfr42I (SacII) (10 U/µl, Fermentas for *HLA-C* | rs1131123), PdiI (NaeI) (10 U/µl, Fermentas for *TSFM* | rs2014886) or a combination of AatII (5 U/µl Fermentas) and BcuI (SpeI) (10 U/µl, Thermo Fisher Scientific) were used for the restriction digestion. Control sequencing was executed by Microsynth Seqlab GmbH with the standard primers T7 and SP6 (Table S6).

Table S1: HTA 2.0 data sets.

Accession number	Arrays (n)	Sample material	Subjects
GSE73080[18]	20	CD4+ cells from the peripheral blood	MS patients
GSE73172[19]	20	CD8+ cells from the peripheral blood	MS patients
GSE81606[20]	20	CD19+ cells from the peripheral blood	MS patients
GSE81603[1]	20	CD14+ cells from the peripheral blood	MS patients
GSE81611[1]	20	CD56+ cells from the peripheral blood	MS patients
GSE63379	32	peripheral blood mononuclear cells	healthy subjects
GSE88887[21, 22]	60	whole blood	healthy subjects
GSE111555	84	whole blood	healthy subjects
GSE111555	32	peripheral blood mononuclear cells	healthy subjects

For each data set used, the GEO database accession number, number of microarrays and information about the tested samples are provided.

Table S2: Assays for SNP genotyping.

**SNP (gene)**	**Assay ID**
rs11074944 (*CLEC16A*)	C__31075379_10
rs3214361 (*CLEC16A*)	Custom
rs3851808 (*EFCAB13*)	C___1578784_10
rs11078928 (*GSDMB*)	C__26309213_10
rs1131123[a] (*HLA-C*)	Custom
rs6897932 (*IL7R*)	C___2025977_10
rs2782 (*NCAPH2*)	C___2251451_20
rs28445040 (*SP140*)	C__25745970_10
rs2014886[a] (*TSFM*)	Custom
rs10783847 (*TSFM*)	C___3188382_10

[a] For technical reasons, rs1131123 was tagged by rs41543814 and rs2014886 was tagged by rs1599932.

Table S3: Antibodies for the assessment of B-cell purity.

**Antibody**	**Source**
CD14-PE, human (clone TÜK4)	Miltenyi Biotec
CD19-FITC, human (clone LT19)	Miltenyi Biotec
CD20-FITC, human (clone LT20)	Miltenyi Biotec
CD3-PerCP, human (clone BW264/56)	Miltenyi Biotec

Table S4: qPCR assays.

Gene	ASE	Assay ID / Custom (sequences 5' → 3')
CLEC16A	All variants	Hs01074744_m1
	Alt. 5' donor site (long exon 11)	Hs01074740_m1
	Alt. last exon (exon 22)	Hs01074751_m1
EFCAB13	All variants	Hs00415984_m1
	Exon 9 & 10 inclusion	Fw: CAAGGCATCCTGGAATGCAT<br>Rev: GATCCTTTGCATCTTGAGAATTTCT<br>Probe: AATATCAACAGTCTTTAGTGCCT
GSDMB	All variants	Hs00938441_m1
	Exon 6 inclusion	Hs00940508_m1
HLA-C	All variants	Hs00740298_g1
	Without intron 2	Fw: GGCCTGCTCCCACTCCAT<br>Rev: CTGTGCCTGGCGCTTGTA<br>Probe: CCGCGGAGTATTGG
IL7R	All variants	Hs00902334_m1
	Exon 6 inclusion	Hs00904815_m1
NCAPH2	Without intron 19	Hs00947246_g1
	Intron 19 retention	Fw: CTTCGAGGTGTGTCGTTCCA<br>Rev: TCCCCCTCCCACGTATCC<br>Probe: CTGCAGCTGGTGAGTAG
SP140	All variants	Hs00916867_m1
	Exon 7 skipping	Fw: GGAGGAGATGCTGAAGATGCA<br>Rev: CATCCCGTTGCTTTCTAGAACTC<br>Probe: CCAGCCTACTACCAGGTG
TSFM	Exon 3 & 4 skipping	Hs00903403_m1
	Exon 3 & 4 inclusion	Fw: GCCGGCGTGCAATGAG<br>Rev: TCTCCTAACGACGCCATTTCTC<br>Probe: TGTGTAGTTTTTGCCTGTACG
	Exon 6 & 7 inclusion	Fw: TGCAAGAAGTTGGATTCAGGAA<br>Rev: GGATTGGTGTGTCGGGATCT<br>Probe: AGGCAGACCCCAGCG
	Exon 6 & 7 skipping	Fw: AGCTGGGCCTGACAGAGAAG<br>Rev: GTGATGAGTCCTCAGCACAATTG<br>Probe: CTCACTCAAGGATCAGTTG

The ASEs covered by the assays are listed. With the *all variant* assays, all transcript isoforms are measured. In case of custom assays, the sequences of forward primer, reverse primer and FAM dye-labeled TaqMan MGB probes were constructed with the Primer Express 3.0 software (Applied Biosystems). The numbering of exons and introns is as specified in Table 1. The assays were purchased from Thermo Fisher Scientific (on TaqMan Array Cards). Alt.: alternative, ASE: alternative splicing event, fw: forward, rev: reverse.

Table S5: Genetic constructs for minigene assays.

Gene	SNP	Genomic position of the inserted sequence	Length	SNP allele[a]
CLEC16A	rs11074944	chr16:11002674-11003705	1032 bp	1023G
CLEC16A	rs11074944	chr16:11002674-11003705	1032 bp	1023A
CLEC16A	rs3214361	chr16:11125579-11126787	1209 bp	327C
CLEC16A	rs3214361	chr16:11125579-11126787	1208 bp	327delC
EFCAB13	rs3851808	chr17:47347408-47348351	944 bp	371C
EFCAB13	rs3851808	chr17:47347408-47348351	944 bp	371T
HLA-C	rs1131123	chr6:31271199-31272071	873 bp	471A
HLA-C	rs1131123	chr6:31271199-31272071	873 bp	471C
NCAPH2	rs2782	chr22:50522617-50523472	856 bp	809T
NCAPH2	rs2782	chr22:50522617-50523472	856 bp	809C
TSFM	rs2014886	chr12:57783215-57784052	838 bp	440C
TSFM	rs2014886	chr12:57783215-57784052	838 bp	440T
TSFM	rs10783847	chr12:57802155-57802774	620 bp	510G
TSFM	rs10783847	chr12:57802155-57802774	620 bp	510A

Sequence regions (according to the GRCh38 reference genome [16]) that were used for the construction of the minigenes. [a] The relative position of the SNP allele within the respective sequence insert is given. bp: base pair.

Table S6: PCR and sequencing primers for the splicing reporter minigene assays.

Primer	Sequence (5' → 3')	Use
PCR_RatInsEx2 (P1)	CCTGCTCATCCTCTGGGAGC	PCR of cDNA samples
PCR_RatInsEx3 (P2)	AGGTCTGAAGGTCACGGGCC	PCR of cDNA samples
PCR_CLEC16A	AGACCTTGAACGCCCACCAT	PCR of cDNA samples
PCR_NCAPH2	CCGGGCTGCTGGGTTATCT	PCR of cDNA samples
PCR_TSFM	GGAGGTGTTCTGCAAGAAGTTGG	PCR of cDNA samples
seq_RatInsEx2	GGATTCTTCTACACACCC	Minigene construct sequencing
seq_RatInsEx3	TCCACCCAGCTCCAGTTG	Minigene construct sequencing
SP6	ATTTAGGTGACACTATAG	PCR product sequencing
T7	TAATACGACTCACTATAGGG	PCR product sequencing

Primer sequences used in the splicing reporter minigene assays. The sequences for seq_RatInsEx2 and seq_RatInsEx3 were obtained from the ExonTrap manual (MoBiTec GmbH), and the sequences for PCR_RatInsEx2 and PCR_RatInsEx3 were taken from the study by Scott *et al.* [23]. SP6 and T7 are standard primers for Microsynth Seqlab's Sanger sequencing service. The other 3 primers were created with the Primer3Plus webtool [24].

Table S7: Softwares, databases and webtools.

Tool	Source
Affymetrix GeneChip Command Console version 4.0	Thermo Fisher Scientific
Custom TaqMan® Assay Design Tool	Thermo Fisher Scientific
Ensembl (release 93 and 104, genome assembly GRCh38)	EMBL-EBI (https://www.ensembl.org/index.html, http://jul2018.archive.ensembl.org/index.html)
ExpressionSuite Software (version 1.3)	Thermo Fisher Scientific
Flowing Software (version 2.5.1)	Turku Bioscience
GEO database	National Center for Biotechnology Information (https://www.ncbi.nlm.nih.gov/geo/)
GTEx portal	Broad Institute of MIT and Harvard, Cambridge (https://www.gtexportal.org/home/)
HGNC human gene nomenclature	HUGO Gene Nomenclature Committee at the EBI (https://www.genenames.org/)
Human Splicing Finder (version 3.1)	Human Splicing Finder - Bioinformatics & Genetics Team, Aix Marseille University (http://www.umd.be/HSF/)
Image Studio™ Lite (version 5.2)	LI-COR Inc.
LDlink (version 3.7 and 3.8)	National Cancer Institute (https://ldlink.nci.nih.gov/?tab=home)
NCBI Genome Remapping Service	National Center for Biotechnology Information (https://www.ncbi.nlm.nih.gov/genome/tools/remap)
Nucleotide Blast	National Center for Biotechnology Information (https://blast.ncbi.nlm.nih.gov/Blast.cgi?PROGRAM=blastn)
POSTAR2	Lu Lab, Tsinghua University (http://postar.ncrnalab.org/)
Primer3Plus	Wageningen University & Research (https://www.bioinformatics.nl/cgi-bin/primer3plus/primer3plus.cgi)
Primer Express (version 3.0)	Thermo Fisher Scientific
R (version 4.0)	The R Foundation
Reactome (database release 71)	The Reactome Project (https://reactome.org/PathwayBrowser/#/R-HSA-72203)
RStudio Desktop (version 1.2.5019)	RStudio Inc.
Single Nucleotide Polymorphism Database (dbSNP build 151)	National Center for Biotechnology Information (https://www.ncbi.nlm.nih.gov/snp/)
TaqMan® Genotyper Software (version 1.6)	Thermo Fisher Scientific
The Human Protein Atlas	SciLifeLab, Solna (http://www.proteinatlas.org/)
Transcriptome Analysis Console Software (version 4.0.1 and 4.0.2)	Thermo Fisher Scientific
UCSC Genome Browser (genome assembly hg38)	University of California Santa Cruz (https://genome.ucsc.edu/cgi-bin/hgGateway)

RESULTS

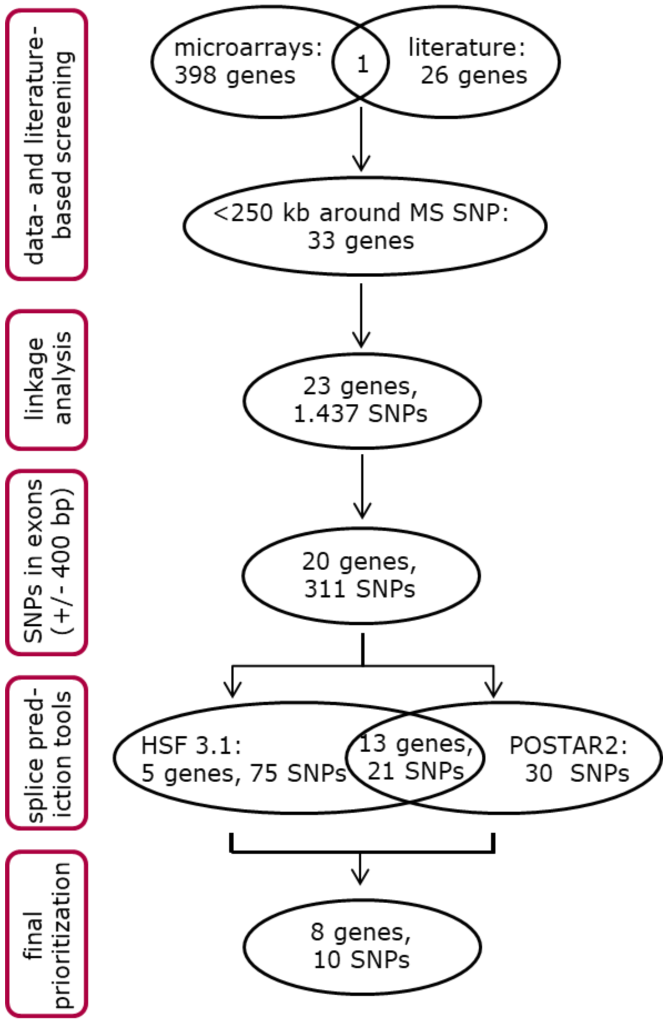

Figure S1: Workflow of the SNP-gene pair selection. Through microarray data and literature-based screening, we identified 425 genes whose splicing pattern presumably differs between MS patients and healthy individuals. Thirty-three of these genes were located less than 250 kb around an MS SNP. By using linkage analyses, we determined a total of 1,437 SNPs located in 23 of the 33 genes, which tended to be inherited together with the MS SNPs. Next, we narrowed down the 1,437 SNPs to 311 SNPs that are located within the exons or the adjacent intronic regions (up to 400 bp away from the exon). We then used the splice prediction tool Human Splicing Finder 3.1 and the POSTAR2 database to screen for SNPs that may regulate the splicing process. In the last step, we assessed whether different splicing events occur in the region around the SNPs in known transcript variants and whether the SNPs also have a potential influence on the protein sequence. In this way, we finally determined 10 SNPs located in 8 different genes. HSF: Human Splicing Finder, MS: Multiple Sclerosis, MS SNP: MS-associated lead SNP from the GWAS [4], SNP: single-nucleotide polymorphism.

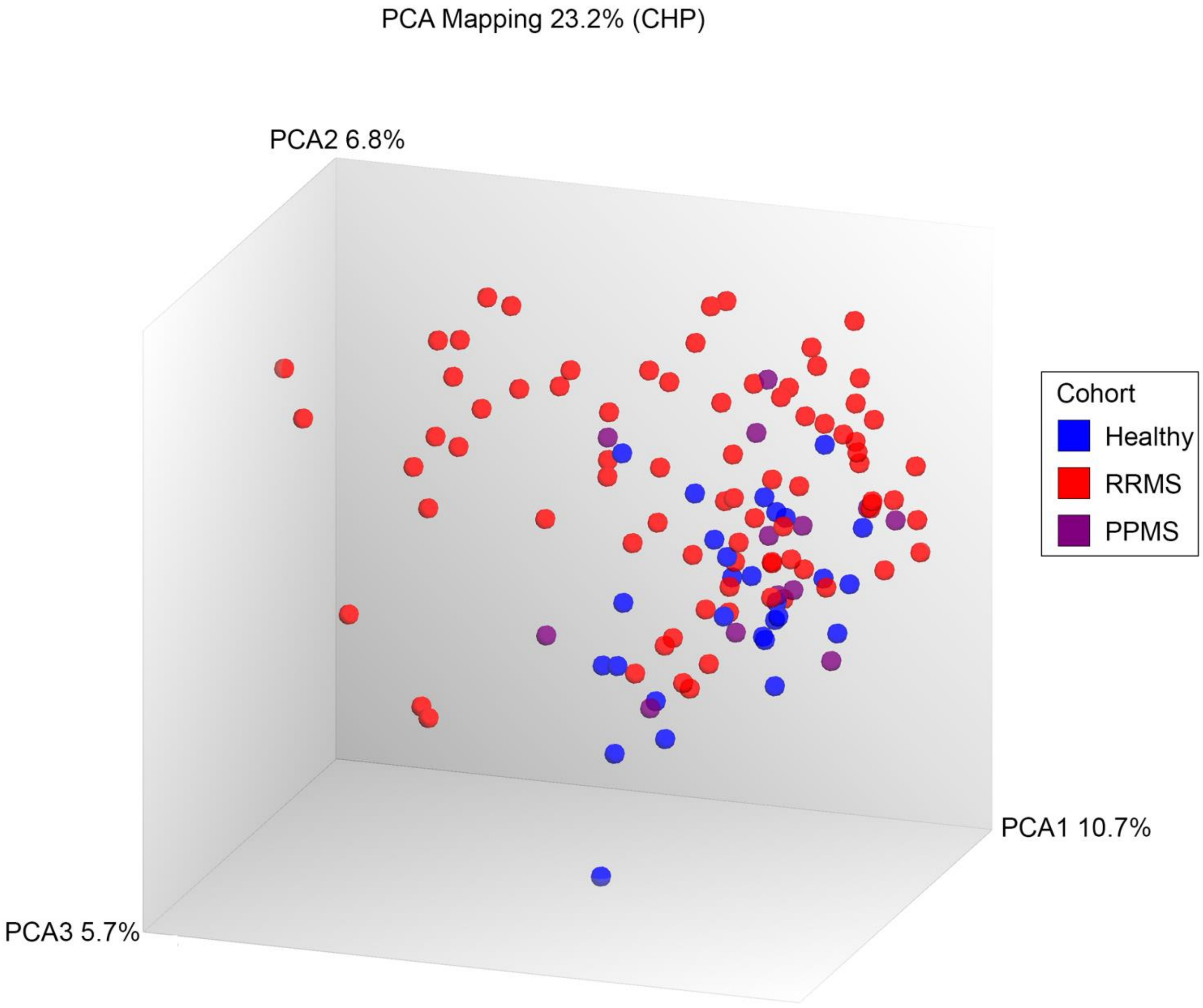

Figure S2: Principal component analysis of B-cell transcriptome profiles from MS patients and healthy subjects. The PCA plot was generated with the TAC software for all 121 microarrays. With three principal components, 23.2% of the variability of the data could be described. A tendency toward cluster formation was found for the samples of the healthy group. No obvious outliers were evident. PCA: principle component analysis, PPMS: primary progressive multiple sclerosis, RRMS: relapsing-remitting multiple sclerosis, TAC: Transcriptome Analysis Console.

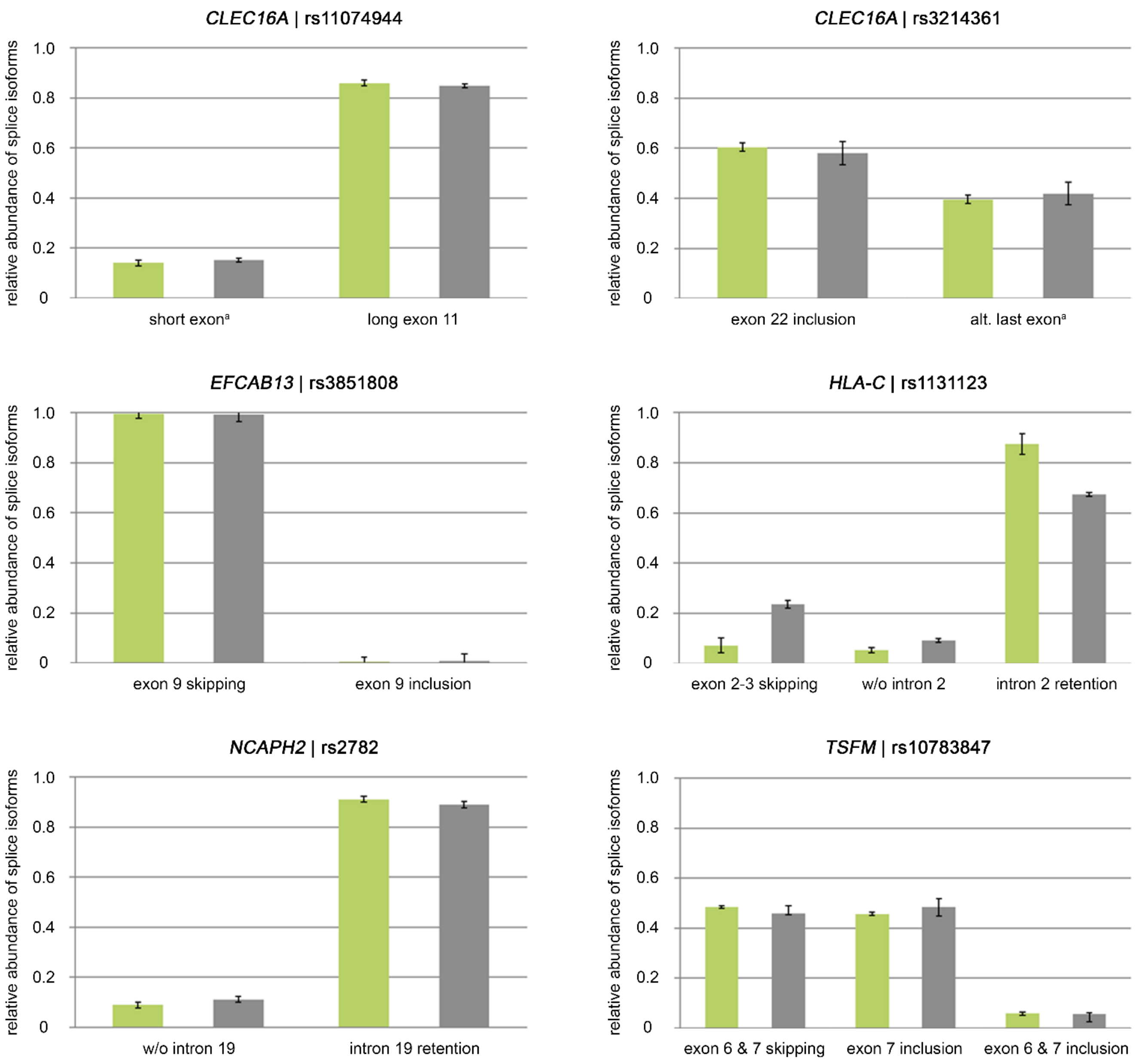

Figure S3: Evaluation of alternative splicing events for six SNP-gene pairs with minigene assays. The relative expression of splice isoforms is shown for the two allelic variants V1 (green) and V2 (gray) of the SNPs. V1 represents the MS risk allele and V2 represents the alternative allele. The numbering of exons and introns is as specified in Table 1. For *HLA-C*, we observed a trend toward preferential intron 2 retention in association with the MS risk allele T of SNP rs1131123. For the other five SNP-gene pairs, we could not confirm a genotype-specific splicing with the minigene assays. Note that no (artificial) exon skipping was observed for the two *CLEC16A* minigene assays. [a] Refers to exon 10 and exon 21 of ENST00000409552. alt.: alternative, w/o: without.

Table S8: Comparison of demographic and clinical data between genotype groups.

Splice SNP	Genotype	MS vs. healthy		RRMS vs. PPMS		Female vs. male		Age in years		Disease duration in years		EDSS score		Relapses in previous year	
	**RA (n)**	**n : n**	**p-value[a]**	**n : n**	**p-value[a]**	**n : n**	**p-value[a]**	**Mean ± SD**	**p-value[b]**	**Mean ± SD**	**p-value[b]**	**Mean ± SD (MV)**	**p-value[b]**	**Mean ± SD**	**p-value[b]**
rs11074944	2 RA (n = 110)	85 : 25	0.7199	75 : 10	0.0844	70 : 40	0.3381	36.4 ± 13.1	0.5154	8.2 ± 6.9	0.8764	3.0 ± 1.6 (10)	0.9067	0.3 ± 0.7	0.3917
	1 RA (n = 11)	8 : 3		5 : 3		9 : 2		39.1 ± 13.0		7.9 ± 3.2		3.1 ± 1.2		0.1 ± 0.4	
	0 RA (n = 0)	—		—		—		—		—		—		—	
rs3214361	genotyping failed														
rs3851808	2 RA (n = 22)	18 : 4	0.8774	15 : 3	0.2838	12 : 10	0.4089	40.1 ± 12.3	0.2629	8.1 ± 5.8	0.9530	3.6 ± 1.8 (3)	0.0595	0.1 ± 0.3	0.7019
	1 RA (n = 53)	38 : 15		31 : 7		36 : 17		36.0 ± 14.5		8.4 ± 7.1		3.0 ± 1.8 (2)		0.5 ± 0.8	
	0 RA (n = 46)	37 : 9		34 : 3		31 : 15		35.7 ± 11.7		8.1 ± 6.6		2.7 ± 1.0 (5)		0.3 ± 0.6	
rs11078928	2 RA (n = 12)	10 : 2	0.5364	8 : 2	0.3899	5 : 7	0.8901	37.3 ± 11.4	0.5872	10.0 ± 6.0	0.7888	2.8 ± 1.3	0.1889	0.6 ± 1.0	0.2354
	1 RA (n = 71)	51 : 20		43 : 8		53 : 18		35.7 ± 13.6		7.7 ± 6.2		2.8 ± 1.4 (5)		0.1 ± 0.4	
	0 RA (n = 38)	32 : 6		29 : 3		21 : 17		38.1 ± 12.8		8.4 ± 7.5		3.4 ± 1.9 (5)		0.6 ± 0.8	
rs1131123*	2 RA (n = 33)	23 : 10	0.6447	16 : 7	0.0318	21 : 12	0.4960	41.3 ± 14.1	**0.0084**	11.4 ± 7.2	0.0261	3.5 ± 2.1 (5)	0.3636	0.3 ± 0.7	0.6308
	1 RA (n = 73)	59 : 14		53 : 6		46 : 27		35.6 ± 13.0		7.1 ± 6.1		2.8 ± 1.3 (4)		0.3 ± 0.6	
	0 RA (n = 15)	11 : 4		11 : 0		12 : 3		31.5 ± 7.5		7.3 ± 6.2		3.2 ± 1.7 (1)		0.5 ± 0.8	
rs6897932	2 RA (n = 70)	55 : 15	0.5986	46 : 9	0.3288	52 : 18	0.2766	36.9 ± 13.6	0.3067	8.6 ± 6.6	0.2581	3.2 ± 1.5 (8)	0.1314	0.3 ± 0.7	0.1866
	1 RA (n = 41)	31 : 10		27 : 4		18 : 23		38.0 ± 13.3		8.3 ± 7.2		2.9 ± 1.8 (2)		0.3 ± 0.6	
	0 RA (n = 10)	7 : 3		7 : 0		9 : 1		29.0 ± 2.9		4.8 ± 1.9		2.1 ± 0.6		0.9 ± 1.1	
rs2782	2 RA (n = 21)	14 : 7	0.3585	12 : 2	0.4031	15 : 6	0.0578	38.6 ± 15.1	0.8757	11.4 ± 7.7	0.5710	3.4 ± 2.1 (2)	0.5644	0.4 ± 0.9	0.0462
	1 RA (n = 65)	51 : 14		46 : 5		47 : 18		35.1 ± 13.1		6.9 ± 6.1		2.9 ± 1.5 (7)		0.4 ± 0.7	
	0 RA (n = 35)	28 : 7		22 : 6		17 : 18		38.2 ± 11.8		8.9 ± 6.5		3.0 ± 1.5 (1)		0.1 ± 0.3	
rs28445040	2 RA (n = 6)	5 : 1	0.3787	4 : 1	0.3388	4 : 2	0.5332	34.0 ± 14.8	0.8490	7.8 ± 6.0	0.1123	2.8 ± 1.6 (1)	0.3760	0.0 ± 0.0	0.4756
	1 RA (n = 48)	39 : 9		32 : 7		29 : 19		37.6 ± 14.6		6.8 ± 6.4		2.8 ± 1.5 (3)		0.3 ± 0.7	
	0 RA (n = 67)	49 : 18		44 : 5		46 : 21		36.1 ± 11.9		9.4 ± 6.7		3.2 ± 1.6 (6)		0.3 ± 0.7	
rs2014886*	2 RA (n = 59)	49 : 10	0.0121	43 : 6	1.0000	41 : 18	0.1871	36.2 ± 13.0	0.8329	8.0 ± 6.1	0.2635	2.9 ± 1.5 (5)	0.4662	0.4 ± 0.8	0.1489
	1 RA (n = 51)	40 : 11		33 : 7		33 : 18		37.9 ± 13.0		7.6 ± 6.6		3.2 ± 1.7 (2)		0.3 ± 0.5	
	0 RA (n = 11)	4 : 7		4 : 0		5 : 6		32.7 ± 14.2		16.5 ± 9.4		2.0 ± N/A (3)		0.0 ± 0.0	
rs10783847	2 RA (n = 60)	49 : 11	0.0193	43 : 6	1.0000	42 : 18	0.1432	36.0 ± 13.1	0.9727	8.0 ± 6.1	0.2635	2.9 ± 1.5 (5)	0.4662	0.4 ± 0.8	0.1489
	1 RA (n = 50)	40 : 10		33 : 7		32 : 18		38.3 ± 12.9		7.6 ± 6.6		3.2 ± 1.7 (2)		0.3 ± 0.5	
	0 RA (n = 11)	4 : 7		4 : 0		5 : 6		32.7 ± 14.2		16.5 ± 9.4		2.0 ± N/A (3)		0.0 ± 0.0	

Descriptive statistics per genotype and p-values are reported for each splice SNP. For age, sex and diagnosis, the analysis was based on all 121 samples. For course of MS, disease duration, degree of disability and number of relapses in the year before the blood sampling, the analysis was based on the subset of 93 samples from patients with MS. Significant differences ($p < 0.01$) are shown in bold. Except that homozygous carrier of the risk allele of SNP rs1131123* were older, no strong differences were noted between the splice SNP genotype groups. * For technical reasons, the designated splice SNP was tagged by a proximal SNP. [a] Fisher's exact tests on allele frequencies were used. [b] F-tests for linear regression were used. —: not available, EDSS: Expanded Disability Status Scale, MS: multiple sclerosis, MV: missing values, n: number, N/A: not available, PPMS: primary progressive MS, RA: risk allele, RRMS: relapsing-remitting MS, SD: standard deviation, SNP: single-nucleotide polymorphism.

Table S9: Examination of alternative splicing events in MS patients vs. healthy controls in the B-cell transcriptome data set.

Gene	PSR / JUC ID	PSR / JUC position (GRCh38)	Healthy (Mean ± SD)	PPMS (Mean ± SD)	RRMS (Mean ± SD)	p-value
CLEC16A	JUC1600049259.hg.1	chr16:10982991-11003074	10.54 ± 0.44	10.68 ± 0.56	10.51 ± 0.64	0.2486
	JUC1600049260.hg.1	chr16:11003257-11020193	5.78 ± 0.67	5.91 ± 0.73	5.70 ± 0.77	0.6585
	JUC1600049272.hg.1	chr16:11003305-11020193	7.24 ± 0.47	7.32 ± 0.50	7.28 ± 0.60	0.9794
	PSR1600149030.hg.1	chr16:11003074-11003257	7.19 ± 0.41	7.61 ± 0.60	7.48 ± 0.51	0.0288
	PSR1600149031.hg.1	chr16:11003258-11003305	9.65 ± 0.29	10.03 ± 0.28	9.96 ± 0.52	**0.0055**
	JUC1600049270.hg.1	chr16:11123946-11125979	6.78 ± 0.52	6.75 ± 0.62	6.77 ± 0.56	0.7862
	JUC1600049273.hg.1	chr16:11126146-11166388	2.99 ± 0.20	3.12 ± 0.16	3.02 ± 0.25	0.3068
	PSR1600149066.hg.1	chr16:11125979-11126009	7.62 ± 0.53	7.85 ± 0.57	7.79 ± 0.62	0.3295
	PSR1600149067.hg.1	chr16:11126010-11126146	7.44 ± 0.36	7.68 ± 0.35	7.57 ± 0.42	0.5266
	PSR1600149068.hg.1	chr16:11126147-11126280	6.42 ± 0.50	6.77 ± 0.22	6.68 ± 0.53	0.0326
	PSR1600149069.hg.1	chr16:11126281-11126344	7.73 ± 0.61	7.93 ± 0.42	7.95 ± 0.49	0.2042
	PSR1600149070.hg.1	chr16:11126345-11126787	7.18 ± 0.64	7.12 ± 0.74	7.31 ± 0.73	0.5997
EFCAB13	JUC1700073474.hg.1	chr17:47345098-47370437	3.45 ± 0.60	3.16 ± 0.78	3.61 ± 0.75	0.3746
	JUC1700073490.hg.1	chr17:47345098-47347808	4.23 ± 0.89	4.48 ± 1.00	4.26 ± 0.86	0.7381
	JUC1700073491.hg.1	chr17:47347951-47361378	4.74 ± 1.25	5.21 ± 1.75	3.96 ± 1.68	0.5450
	JUC1700073492.hg.1	chr17:47361521-47370437	6.52 ± 2.34	7.42 ± 2.26	7.35 ± 2.32	0.6541
	JUC1700073495.hg.1	chr17:47345098-47361378	4.58 ± 1.14	5.09 ± 1.33	4.69 ± 1.12	0.6164
	PSR1700202809.hg.1	chr17:47347808-47347951	9.33 ± 0.72	9.65 ± 0.75	9.38 ± 0.89	0.5120
	PSR1700202811.hg.1	chr17:47361378-47361521	11.02 ± 2.36	12.29 ± 2.42	11.38 ± 2.64	0.6927
GSDMB	JUC1700064890.hg.1	chr17:39906987-39908176	3.89 ± 0.47	3.81 ± 0.43	3.93 ± 0.53	0.8893
	JUC1700064891.hg.1	chr17:39908214-39908958	3.74 ± 0.90	3.69 ± 0.62	3.68 ± 0.75	0.5540
	JUC1700064895.hg.1	chr17:39906271-39908176	2.88 ± 0.34	2.97 ± 0.35	2.88 ± 0.37	0.4016
	JUC1700064896.hg.1	chr17:39908226-39908958	5.60 ± 0.68	5.38 ± 0.56	5.72 ± 0.63	0.7023
	JUC1700064897.hg.1	chr17:39906271-39908958	3.62 ± 0.67	3.74 ± 0.42	4.06 ± 0.50	0.1001
	JUC1700064898.hg.1	chr17:39906987-39908958	5.69 ± 0.63	5.85 ± 0.76	5.86 ± 0.66	0.4619
	PSR1700183459.hg.1	chr17:39908176-39908214	9.91 ± 0.89	10.91 ± 1.11	9.97 ± 1.54	0.3003
HLA-C[a]	PSR0600200977.hg.1	chr6:31271603-31271868	15.90 ± 0.79	15.87 ± 0.53	15.84 ± 0.83	0.6744
IL7R	JUC0500049289.hg.1	chr5:35873648-35874449	11.99 ± 1.63	9.57 ± 0.84	10.51 ± 1.68	**1.6e-05**
	JUC0500049290.hg.1	chr5:35874542-35875512	11.07 ± 2.04	7.97 ± 0.87	9.45 ± 2.12	**4.5e-05**
	JUC0500049293.hg.1	chr5:35873648-35875512	5.26 ± 1.00	4.15 ± 0.61	4.52 ± 1.15	**0.0044**
	PSR0500148308.hg.1	chr5:35874449-35874542	10.50 ± 1.83	8.10 ± 0.65	9.22 ± 1.77	**4.7e-05**

Gene	PSR / JUC ID	PSR / JUC position (GRCh38)	Healthy (Mean ± SD)	PPMS (Mean ± SD)	RRMS (Mean ± SD)	p-value
NCAPH2	JUC2200052280.hg.1	chr22:50522922-50523017	6.26 ± 0.46	6.16 ± 0.23	6.42 ± 0.38	0.1698
	JUC2200052281.hg.1	chr22:50523166-50523235	5.44 ± 0.43	5.69 ± 0.79	5.46 ± 0.65	0.3229
	JUC2200052288.hg.1	chr22:50522922-50523080	2.71 ± 0.14	2.71 ± 0.22	2.71 ± 0.20	0.7109
	PSR2200155519.hg.1	chr22:50523017-50523079	4.10 ± 0.23	4.07 ± 0.21	4.10 ± 0.38	0.7889
	PSR2200155520.hg.1	chr22:50523080-50523166	5.99 ± 0.50	5.99 ± 0.52	5.86 ± 0.52	0.3654
	PSR2200155521.hg.1	chr22:50523167-50523234	8.30 ± 0.40	8.12 ± 0.35	8.30 ± 0.46	0.4553
	PSR2200155522.hg.1	chr22:50523235-50523375	4.14 ± 0.32	4.14 ± 0.36	4.00 ± 0.37	0.5466
	PSR2200155523.hg.1	chr22:50523376-50523470	4.11 ± 0.37	3.88 ± 0.45	3.99 ± 0.44	0.2440
SP140	JUC0200064632.hg.1	chr2:230245080-230245863	14.22 ± 0.81	13.97 ± 0.97	14.51 ± 1.01	0.3333
	JUC0200064633.hg.1	chr2:230245940-230247916	14.55 ± 1.11	14.42 ± 1.22	14.86 ± 1.04	0.3546
	JUC0200064653.hg.1	chr2:230245071-230245863	8.07 ± 1.44	7.75 ± 1.93	8.26 ± 1.63	0.1490
	JUC0200064654.hg.1	chr2:230245940-230248885	5.33 ± 0.88	4.67 ± 0.93	5.53 ± 1.03	0.0460
	JUC0200064656.hg.1	chr2:230245080-230247916	8.62 ± 1.25	9.25 ± 1.19	8.77 ± 1.24	0.4886
	JUC0200064664.hg.1	chr2:230245080-230245864	12.91 ± 0.70	12.71 ± 1.35	13.34 ± 1.10	0.2447
	PSR0200178948.hg.1	chr2:230245864-230245913	15.15 ± 0.87	15.65 ± 1.06	15.66 ± 1.09	0.1276
	PSR0200178949.hg.1	chr2:230245914-230245940	12.09 ± 0.84	11.99 ± 1.16	12.48 ± 1.05	0.3381
TSFM	JUC1200073939.hg.1	chr12:57783283-57783615	2.55 ± 0.17	2.54 ± 0.13	2.58 ± 0.20	0.2360
	JUC1200073940.hg.1	chr12:57783652-57783941	4.56 ± 0.48	4.78 ± 0.33	4.66 ± 0.58	0.4886
	JUC1200073941.hg.1	chr12:57784170-57786163	5.25 ± 0.51	5.06 ± 0.49	5.18 ± 0.63	0.7915
	JUC1200073943.hg.1	chr12:57783283-57786163	4.29 ± 0.51	4.43 ± 0.55	4.38 ± 0.56	0.6226
	JUC1200073944.hg.1	chr12:57783652-57786163	7.22 ± 0.75	7.27 ± 0.60	7.27 ± 0.94	0.9535
	PSR1200200787.hg.1	chr12:57783615-57783652	13.39 ± 0.54	13.31 ± 0.46	13.27 ± 0.73	0.4849
	PSR1200200788.hg.1	chr12:57783941-57784170	5.97 ± 0.55	5.82 ± 0.58	5.57 ± 0.56	**0.0066**
	JUC1200073951.hg.1	chr12:57793073-57802555	3.72 ± 0.61	3.41 ± 0.69	3.92 ± 0.74	0.0673
	JUC1200073952.hg.1	chr12:57793073-57802174	5.31 ± 0.58	5.16 ± 0.31	5.37 ± 0.61	0.7118
	JUC1200073953.hg.1	chr12:57802264-57802555	5.66 ± 0.45	5.33 ± 0.33	5.55 ± 0.47	0.1497
	JUC1200073954.hg.1	chr12:57793073-57807864	3.50 ± 0.39	3.71 ± 0.57	3.46 ± 0.43	0.0693
	PSR1200200801.hg.1	chr12:57802174-57802264	6.19 ± 0.47	6.38 ± 0.46	6.33 ± 0.43	0.1273
	PSR1200200802.hg.1	chr12:57802555-57802631	6.06 ± 0.59	6.04 ± 0.69	5.84 ± 0.56	0.4054
	PSR1200200803.hg.1	chr12:57802632-57802774	3.26 ± 0.40	3.37 ± 0.49	3.20 ± 0.43	0.8234

Shown are Tukey biweight means and standard deviations of log2 signal intensities for the levels of exons and exon-exon junctions that belong to the ASEs of the ten prioritized SNP-gene pairs (Mean ± SD). Significant expression differences ($p < 0.01$) are shown in bold. In MS patients, especially in PPMS patients, the levels of exon 6 of *IL7R* were significantly lower, indicating that this exon is skipped more frequently as compared to healthy controls. The genomic region covered by the PSR / JUC probe sets according to genome assembly GRCh38 is given (PSR / JUC position). [a] Since *HLA-C* intron 2 exclusion was not

represented by probe sets of the Clariom D microarrays, only data for intron 2 retention is shown. ASE: alternative splicing event, JUC: probe set for exon-exon junction, MS: multiple sclerosis, N/A: not available, PPMS: primary progressive MS, PSR: probe set for exon fragment (probe selection region), RRMS: relapsing-remitting MS.

Table S10: Examination of alternative splicing events in relation to splice SNP genotypes in the B-cell transcriptome data set.

Gene	SNP	MS RA	PSR / JUC ID	PSR / JUC position (GRCh38)	0 RA (Mean ± SD)	1 RA (Mean ± SD)	2 RA (Mean ± SD)	p-value
CLEC16A	rs11074944	G	JUC1600049259.hg.1	chr16:10982991-11003074	N/A	10.77 ± 0.48	10.52 ± 0.60	0.3034
			JUC1600049260.hg.1	chr16:11003257-11020193	N/A	5.77 ± 0.85	5.73 ± 0.73	0.9382
			JUC1600049272.hg.1	chr16:11003305-11020193	N/A	7.46 ± 0.43	7.26 ± 0.57	0.7743
			PSR1600149030.hg.1	chr16:11003074-11003257	N/A	7.41 ± 0.65	7.42 ± 0.50	0.5873
			PSR1600149031.hg.1	chr16:11003258-11003305	N/A	10.04 ± 0.35	9.86 ± 0.49	0.9622
CLEC16A	rs3214361	C	genotyping failed					
EFCAB13	rs3851808	C	JUC1700073474.hg.1	chr17:47345098-47370437	3.90 ± 0.80	3.45 ± 0.57	3.18 ± 0.57	**0.0001**
			JUC1700073490.hg.1	chr17:47345098-47347808	4.02 ± 0.73	4.38 ± 0.82	4.72 ± 1.10	**0.0047**
			JUC1700073491.hg.1	chr17:47347951-47361378	3.13 ± 0.48	5.01 ± 1.38	6.73 ± 0.97	**5.8e-23**
			JUC1700073492.hg.1	chr17:47361521-47370437	3.23 ± 2.10	7.49 ± 1.33	8.92 ± 1.08	**2.7e-19**
			JUC1700073495.hg.1	chr17:47345098-47361378	3.76 ± 0.92	5.01 ± 1.01	5.85 ± 0.83	**7.1e-11**
			PSR1700202809.hg.1	chr17:47347808-47347951	9.49 ± 0.71	9.17 ± 0.82	9.66 ± 1.00	0.0341
			PSR1700202811.hg.1	chr17:47361378-47361521	7.32 ± 2.42	11.55 ± 1.32	13.53 ± 0.81	**3.1e-20**
GSDMB	rs11078928	C	JUC1700064890.hg.1	chr17:39906987-39908176	4.07 ± 0.58	3.92 ± 0.45	3.51 ± 0.33	**0.0024**
			JUC1700064891.hg.1	chr17:39908214-39908958	3.97 ± 0.94	3.66 ± 0.63	3.31 ± 0.42	**0.0003**
			JUC1700064895.hg.1	chr17:39906271-39908176	2.87 ± 0.32	2.91 ± 0.38	2.89 ± 0.37	0.5769
			JUC1700064896.hg.1	chr17:39908226-39908958	5.65 ± 0.71	5.71 ± 0.60	5.45 ± 0.51	0.3751
			JUC1700064897.hg.1	chr17:39906271-39908958	3.85 ± 0.57	3.96 ± 0.53	3.96 ± 0.56	0.7700
			JUC1700064898.hg.1	chr17:39906987-39908958	5.77 ± 0.77	5.86 ± 0.63	5.81 ± 0.52	0.9487
			PSR1700183459.hg.1	chr17:39908176-39908214	10.47 ± 1.44	10.13 ± 1.04	7.94 ± 0.97	**1.2e-09**
HLA-C[a]	rs1131123*	T	PSR0600200977.hg.1	chr6:31271603-31271868	14.93 ± 0.93	15.85 ± 0.68	16.09 ± 0.70	**5.8e-06**
IL7R	rs6897932	C	JUC0500049289.hg.1	chr5:35873648-35874449	11.10 ± 0.91	10.66 ± 1.81	10.69 ± 1.81	0.9105
			JUC0500049290.hg.1	chr5:35874542-35875512	10.25 ± 1.26	9.43 ± 2.04	9.61 ± 2.35	0.8431
			JUC0500049293.hg.1	chr5:35873648-35875512	3.90 ± 0.95	4.62 ± 1.08	4.75 ± 1.14	0.1277
			PSR0500148308.hg.1	chr5:35874449-35874542	9.00 ± 0.58	9.04 ± 1.90	9.69 ± 1.89	0.2265
NCAPH2	rs2782	T	JUC2200052280.hg.1	chr22:50522922-50523017	6.30 ± 0.39	6.40 ± 0.42	6.35 ± 0.32	0.6437
			JUC2200052281.hg.1	chr22:50523166-50523235	5.56 ± 0.73	5.42 ± 0.56	5.52 ± 0.66	0.7605
			JUC2200052288.hg.1	chr22:50522922-50523080	2.76 ± 0.18	2.72 ± 0.20	2.63 ± 0.14	0.0448
			PSR2200155519.hg.1	chr22:50523017-50523079	4.06 ± 0.28	4.17 ± 0.34	3.98 ± 0.40	0.1562
			PSR2200155520.hg.1	chr22:50523080-50523166	5.89 ± 0.49	5.88 ± 0.53	5.97 ± 0.53	0.7720
			PSR2200155521.hg.1	chr22:50523167-50523234	8.29 ± 0.37	8.27 ± 0.46	8.31 ± 0.47	0.7614
			PSR2200155522.hg.1	chr22:50523235-50523375	4.05 ± 0.34	3.97 ± 0.37	4.05 ± 0.35	0.4762
			PSR2200155523.hg.1	chr22:50523376-50523470	3.85 ± 0.44	4.06 ± 0.44	4.06 ± 0.40	0.4472

Gene	SNP	MS RA	PSR / JUC ID	PSR / JUC position (GRCh38)	0 RA (Mean ± SD)	1 RA (Mean ± SD)	2 RA (Mean ± SD)	p-value
SP140	rs28445040	T	JUC0200064632.hg.1	chr2:230245080-230245863	14.87 ± 0.40	13.80 ± 0.39	11.36 ± 0.88	**1.6e-43**
			JUC0200064633.hg.1	chr2:230245940-230247916	15.37 ± 0.73	13.95 ± 0.74	12.63 ± 0.97	**1.4e-20**
			JUC0200064653.hg.1	chr2:230245071-230245863	9.01 ± 0.97	7.40 ± 0.94	3.17 ± 0.21	**2.0e-30**
			JUC0200064654.hg.1	chr2:230245940-230248885	5.81 ± 0.91	4.92 ± 0.83	4.22 ± 0.81	**1.8e-08**
			JUC0200064656.hg.1	chr2:230245080-230247916	7.97 ± 0.70	9.78 ± 0.68	11.24 ± 0.61	**1.4e-31**
			JUC0200064664.hg.1	chr2:230245080-230245864	13.72 ± 0.49	12.43 ± 0.47	9.71 ± 0.80	**3.2e-40**
			PSR0200178948.hg.1	chr2:230245864-230245913	16.05 ± 1.02	15.04 ± 0.83	14.26 ± 0.28	**1.8e-07**
			PSR0200178949.hg.1	chr2:230245914-230245940	12.97 ± 0.93	11.80 ± 0.71	10.88 ± 0.55	**1.5e-11**
TSFM	rs2014886*	C	JUC1200073939.hg.1	chr12:57783283-57783615	2.66 ± 0.24	2.55 ± 0.16	2.58 ± 0.20	0.2915
			JUC1200073940.hg.1	chr12:57783652-57783941	4.33 ± 0.53	4.70 ± 0.57	4.68 ± 0.51	0.4424
			JUC1200073941.hg.1	chr12:57784170-57786163	5.54 ± 0.63	5.37 ± 0.62	4.97 ± 0.48	**0.0012**
			JUC1200073943.hg.1	chr12:57783283-57786163	4.10 ± 0.64	4.29 ± 0.41	4.55 ± 0.60	0.0206
			JUC1200073944.hg.1	chr12:57783652-57786163	7.22 ± 0.71	7.01 ± 0.84	7.48 ± 0.87	0.0309
			PSR1200200787.hg.1	chr12:57783615-57783652	13.33 ± 0.87	13.26 ± 0.49	13.35 ± 0.74	0.3255
			PSR1200200788.hg.1	chr12:57783941-57784170	6.65 ± 0.54	5.78 ± 0.53	5.46 ± 0.50	**5.0e-07**
TSFM	rs10783847	G	JUC1200073951.hg.1	chr12:57793073-57802555	3.65 ± 0.68	3.80 ± 0.67	3.85 ± 0.76	0.3012
			JUC1200073952.hg.1	chr12:57793073-57802174	5.21 ± 0.73	5.28 ± 0.61	5.36 ± 0.52	0.5333
			JUC1200073953.hg.1	chr12:57802264-57802555	5.37 ± 0.53	5.57 ± 0.46	5.56 ± 0.44	0.8223
			JUC1200073954.hg.1	chr12:57793073-57807864	3.50 ± 0.38	3.64 ± 0.44	3.37 ± 0.42	**0.0022**
			PSR1200200801.hg.1	chr12:57802174-57802264	6.08 ± 0.30	6.27 ± 0.51	6.37 ± 0.40	0.0585
			PSR1200200802.hg.1	chr12:57802555-57802631	6.00 ± 0.67	5.80 ± 0.50	5.98 ± 0.64	0.7043
			PSR1200200803.hg.1	chr12:57802632-57802774	2.85 ± 0.28	3.15 ± 0.38	3.33 ± 0.45	**0.0011**

Shown are Tukey biweight means and standard deviations of log2 signal intensities for the levels of exons and exon-exon junctions that belong to ASEs of the ten prioritized SNP-gene pairs (Mean ± SD). Significant expression differences ($p < 0.01$) in the data between the genotype groups are shown in bold. Differential expression of ASE-specific exons and exon-exon junctions provided evidence of genotype-dependent pre-mRNA splicing for *EFCAB13* (rs3851808), *GSDMB* (rs11078928), *HLA-C* (rs1131123*), *SP140* (rs28445040) and *TSFM* (rs2014886*, rs10783847). The genomic region covered by the PSR / JUC probe sets according to genome assembly GRCh38 is given (PSR / JUC position). [*] For technical reasons, the designated splice SNP was tagged by a proximal SNP. [a] Since *HLA-C* intron 2 exclusion was not represented by probe sets of the Clariom D microarrays, only data for intron 2 retention is shown. ASE: alternative

splicing event, JUC: probe set for exon-exon junction, MS: multiple sclerosis, N/A: not available, PSR: probe set for exon fragment (probe selection region), RA: risk allele, SNP: single-nucleotide polymorphism.

Table S11: Differential expression of transcript isoforms in comparison of MS patients and healthy controls in the qPCR data set.

Gene	Group (n)	ASE 1				ASE 2				All variants		
		Type	MV	Mean ± SD	p-value	Type	MV	Mean ± SD	p-value	MV	Mean ± SD	p-value
CLEC16A	Healthy (n = 25)	long exon	0	75.54 ± 20.26	0.7001	short exon[a,c]	0	220.80 ± 68.99	0.1023	0	296.33 ± 80.37	0.2279
	PPMS (n = 11)	11	0	82.60 ± 20.81			0	203.19 ± 39.85		0	285.79 ± 48.08	
	RRMS (n = 73)		0	75.20 ± 29.92			0	191.65 ± 56.97		0	266.85 ± 78.12	
CLEC16A	Healthy (n = 25)	exon 22	0	132.90 ± 52.02	0.0125	alt. last	0	163.43 ± 75.39	0.4391	0	296.33 ± 80.37	0.2279
	PPMS (n = 11)		0	148.62 ± 60.19		exon[a,c]	0	137.17 ± 73.09		0	285.79 ± 48.08	
	RRMS (n = 73)		0	108.27 ± 47.59			0	158.58 ± 47.46		0	266.85 ± 78.12	
EFCAB13	Healthy (n = 25)	exon 9 & 10	5	19.77 ± 20.13	0.9909	exon 9 & 10	5	62.99 ± 33.15	0.9790	0	82.76 ± 35.95	0.9655
	PPMS (n = 11)	inclusion	2	19.41 ± 27.52		skipping[a]	2	64.99 ± 29.38		0	84.40 ± 39.80	
	RRMS (n = 73)		11	20.45 ± 32.60			11	64.58 ± 36.98		0	85.03 ± 36.92	
GSDMB	Healthy (n = 25)	exon 6	0	49.26 ± 50.89	0.8156	exon 6	0	981.62 ± 850.88	0.2145	0	1030.88 ± 865.17	0.2254
	PPMS (n = 11)	inclusion	0	61.04 ± 51.63		skipping[a]	0	1079.50 ± 779.83		0	1140.54 ± 781.33	
	RRMS (n = 73)		0	53.02 ± 50.91			0	741.31 ± 733.69		0	794.33 ± 753.05	
HLA-C	Healthy (n = 25)	without	0	5673.15 ± 4759.72	0.5373	intron 2		N/A[d]				
	PPMS (n = 11)	intron 2	0	4423.68 ± 3353.48		retention						
	RRMS (n = 73)		0	4786.48 ± 3489.30								
IL7R	Healthy (n = 25)	exon 6	0	62.06 ± 35.36	**0.0015**	exon 6	0	47.13 ± 73.10	0.0144	0	109.19 ± 88.86	**0.0004**
	PPMS (n = 11)	inclusion	0	17.38 ± 12.75		skipping[a]	0	18.92 ± 24.89		0	36.30 ± 22.80	
	RRMS (n = 73)		0	39.26 ± 37.44			0	19.60 ± 24.19		0	58.86 ± 49.67	
NCAPH2[b]	Healthy (n = 25)	without	0	149.13 ± 42.44	0.0495	intron 19	0	73.32 ± 40.33	0.0594	0	222.45 ± 63.26	0.0196
	PPMS (n = 11)	intron 19	0	153.54 ± 43.05		retention	0	66.16 ± 30.98		0	219.71 ± 55.25	
	RRMS (n = 73)		0	126.43 ± 49.97			0	53.01 ± 37.86		0	179.44 ± 77.35	
SP140	Healthy (n = 25)	exon 7	0	918.50 ± 411.83	0.7776	exon 7	0	88.46 ± 84.25	0.0996	0	1006.96 ± 451.05	0.8054
	PPMS (n = 11)	inclusion[a]	0	941.18 ± 510.46		skipping	0	153.27 ± 173.82		0	1094.45 ± 661.84	
	RRMS (n = 73)		0	1020.41 ± 744.08			0	89.07 ± 78.93		0	1109.49 ± 734.91	
TSFM[b]	Healthy (n = 25)	exon 3 & 4	19	0.12 ± 0.24	0.7139	exon 3 & 4	0	61.40 ± 20.13	0.8845	19	61.52 ± 20.14	0.8901
	PPMS (n = 11)	inclusion	9	0.10 ± 0.20		skipping	0	60.21 ± 13.21		9	60.31 ± 13.25	
	RRMS (n = 73)		48	0.16 ± 0.33			0	59.45 ± 16.51		48	59.61 ± 16.56	
TSFM[b]	Healthy (n = 25)	exon 6 & 7	6	1.02 ± 0.95	0.8438	exon 6 & 7	5	0.51 ± 0.63	**0.0054**	9	1.54 ± 1.08	0.6833
	PPMS (n = 11)	inclusion	3	1.32 ± 0.58		skipping	2	0.74 ± 0.89		3	2.06 ± 1.24	
	RRMS (n = 73)		9	1.25 ± 2.12			12	0.29 ± 0.29		19	1.54 ± 2.15	

Means and standard deviations of normalized qPCR data (in linear scale, 109 B-cell RNA samples) of specific transcript isoforms (Mean ± SD). The number of samples in which the corresponding transcript could not be detected and for which C_T values were thus imputed is indicated (MV). Significant expression differences ($p < 0.01$) are shown in bold. [a] Mean ± SD of the transcript isoform expression was calculated from the differences in the data of two assays. [b] The data for *all variants* were calculated by the sum of two assays capturing distinct splicing isoforms. [c] Exon related to ENST00000409552. [d] No evaluation possible,

as assay Hs00740298_g1 did not provide valid data, possibly due to sensitivity to *HLA-C* subtypes. ASE: alternative splicing event, MS: multiple sclerosis, MV: missing values, n: number, N/A: not available, PPMS: primary progressive MS, RA: risk allele, RRMS: relapsing-remitting MS, SNP: single-nucleotide polymorphism.

Table S12: Differential expression of transcript isoforms in relation to splice SNP genotypes in the qPCR data set.

Gene, SNP	RA (n)	ASE 1				ASE 2				All variants		
		Type	MV	Mean ± SD	p-value	Type	MV	Mean ± SD	p-value	MV	Mean ± SD	p-value
CLEC16A, rs11074944	2 RA (n = 100)	long exon 11	0	74.24 ± 26.50	0.0206	short exon[a,c]	0	198.89 ± 59.59	0.7208	0	273.12 ± 76.21	0.2772
	1 RA (n = 9)		0	95.93 ± 26.86			0	206.31 ± 58.88		0	302.24 ± 81.38	
	0 RA (n = 0)		N/A	N/A			N/A	N/A		N/A	N/A	
CLEC16A, rs3214361	genotyping failed											
EFCAB13, rs3851808	2 RA (n = 18)	exon 9 & 10 inclusion	0	59.34 ± 42.59	**3.4e-14**	exon 9 & 10 skipping[a]	0	60.20 ± 50.41	0.7670	0	119.54 ± 34.58	**5.6e-08**
	1 RA (n = 49)		1	21.29 ± 20.96			1	65.65 ± 35.61		0	86.94 ± 35.13	
	0 RA (n = 42)		17	2.13 ± 3.74			17	64.37 ± 26.63		0	66.50 ± 26.83	
GSDMB, rs11078928	2 RA (n = 12)	exon 6 inclusion	0	1.19 ± 2.38	**5.4e-06**	exon 6 skipping[a]	0	437.22 ± 438.34	0.3831	0	438.42 ± 439.12	0.2562
	1 RA (n = 64)		0	50.28 ± 37.73			0	910.55 ± 739.82		0	960.83 ± 748.03	
	0 RA (n = 33)		0	76.99 ± 64.97			0	818.45 ± 887.09		0	895.44 ± 911.94	
HLA-C, rs1131123*	2 RA (n = 29)	without intron 2	0	4851.27 ± 3601.61	0.8859	intron 2 retention	N/A[d]					
	1 RA (n = 65)		0	4989.49 ± 3830.44								
	0 RA (n = 15)		0	4993.27 ± 4210.72								
IL7R, rs6897932	2 RA (n = 64)	exon 6 inclusion	0	40.71 ± 38.50	0.8838	exon 6 skipping[a]	0	28.91 ± 48.65	0.4595	0	69.62 ± 69.81	0.6850
	1 RA (n = 37)		0	46.77 ± 38.83			0	20.81 ± 30.96		0	67.58 ± 57.03	
	0 RA (n = 8)		0	34.17 ± 10.20			0	24.63 ± 29.54		0	58.79 ± 31.54	
NCAPH2[b], rs2782	2 RA (n = 18)	without intron 19	0	147.47 ± 54.08	0.7155	intron 19 retention	0	60.56 ± 33.39	0.8392	0	208.03 ± 82.01	0.7319
	1 RA (n = 60)		0	124.46 ± 46.71			0	57.15 ± 44.25		0	181.62 ± 76.70	
	0 RA (n = 31)		0	145.94 ± 46.21			0	61.65 ± 28.97		0	207.60 ± 63.36	
SP140, rs28445040	2 RA (n = 5)	exon 7 inclusion[a]	0	627.67 ± 84.40	0.0183	exon 7 skipping	0	272.56 ± 129.84	**3.2e-18**	0	900.23 ± 162.91	0.2090
	1 RA (n = 41)		0	847.84 ± 372.47			0	156.19 ± 94.40		0	1004.04 ± 454.92	
	0 RA (n = 63)		0	1109.62 ± 791.05			0	41.80 ± 28.00		0	1151.41 ± 795.60	
TSFM[b], rs2014886*	2 RA (n = 53)	exon 3 & 4 inclusion	47	0.02 ± 0.08	**1.2e-05**	exon 3 & 4 skipping	0	61.85 ± 17.16	0.2610	47	61.87 ± 17.17	0.2954
	1 RA (n = 45)		24	0.24 ± 0.37			0	58.50 ± 17.47		24	58.74 ± 17.57	
	0 RA (n = 11)		5	0.35 ± 0.44			0	56.96 ± 14.32		5	57.31 ± 14.34	
TSFM[b], rs10783847	2 RA (n = 54)	exon 6 & 7 inclusion	9	1.45 ± 2.45	0.1827	exon 6 & 7 skipping[a]	12	0.35 ± 0.33	0.5245	19	1.80 ± 2.48	0.2672
	1 RA (n = 44)		6	0.98 ± 0.65			5	0.43 ± 0.64		9	1.41 ± 0.96	
	0 RA (n = 11)		3	0.90 ± 0.76			2	0.41 ± 0.49		3	1.31 ± 0.94	

Means and standard deviations of normalized qPCR data (in linear scale, 109 B-cell RNA samples) of specific transcript isoforms (Mean ± SD). The number of samples in which the corresponding transcript could not be detected and for which C_T values were thus imputed is indicated (MV). Significant expression differences ($p < 0.01$) in the data between the genotype groups are shown in bold. For *EFCAB13*, *GSDMB*, *SP140* and *TSFM*, we observed differential expression of ASE-specific transcript isoforms, which points to genotype-dependent pre-mRNA splicing. * For technical reasons, the designated splice SNP was

tagged by a proximal SNP. [a] Mean ± SD of the transcript isoform expression was calculated from the differences in the data of two assays. [b] The data for *all variants* were calculated by the sum of two assays capturing distinct splicing isoforms. [c] Exon related to ENST00000409552. [d] No evaluation possible, as assay Hs00740298_g1 did not provide valid data, possibly due to sensitivity to *HLA-C* subtypes. ASE: alternative splicing event, MS: multiple sclerosis, MV: missing values, n: number, N/A: not available, PPMS: primary progressive MS, RA: risk allele, RRMS: relapsing-remitting MS, SNP: single-nucleotide polymorphism.

References

1. Koczan D, Fitzner B, Zettl UK, Hecker M. Microarray data of transcriptome shifts in blood cell subsets during S1P receptor modulator therapy. Sci Data. 2018;5:180145. doi:10.1038/sdata.2018.145.
2. Romero JP, Muniategui A, Miguel FJ de, Aramburu A, Montuenga L, Pio R, Rubio A. EventPointer: an effective identification of alternative splicing events using junction arrays. BMC Genomics. 2016;17:467. doi:10.1186/s12864-016-2816-x.
3. Hecker M, Rüge A, Putscher E, Boxberger N, Rommer PS, Fitzner B, Zettl UK. Aberrant expression of alternative splicing variants in multiple sclerosis - A systematic review. Autoimmun Rev. 2019;18:721–32. doi:10.1016/j.autrev.2019.05.010.
4. International Multiple Sclerosis Genetics Consortium. Multiple sclerosis genomic map implicates peripheral immune cells and microglia in susceptibility. Science. 2019;365:eaav7188. doi:10.1126/science.aav7188.
5. Machiela MJ, Chanock SJ. LDlink: a web-based application for exploring population-specific haplotype structure and linking correlated alleles of possible functional variants. Bioinformatics. 2015;31:3555–7. doi:10.1093/bioinformatics/btv402.
6. Desmet F-O, Hamroun D, Lalande M, Collod-Béroud G, Claustres M, Béroud C. Human Splicing Finder: an online bioinformatics tool to predict splicing signals. Nucleic Acids Res. 2009;37:e67. doi:10.1093/nar/gkp215.
7. Zhu Y, Xu G, Yang YT, Xu Z, Chen X, Shi B, et al. POSTAR2: deciphering the post-transcriptional regulatory logics. Nucleic Acids Res. 2019;47:D203–11. doi:10.1093/nar/gky830.
8. Fu X-D, Ares M. Context-dependent control of alternative splicing by RNA-binding proteins. Nat Rev Genet. 2014;15:689–701. doi:10.1038/nrg3778.
9. Long JC, Caceres JF. The SR protein family of splicing factors: master regulators of gene expression. Biochem J. 2009;417:15–27. doi:10.1042/BJ20081501.
10. Geuens T, Bouhy D, Timmerman V. The hnRNP family: insights into their role in health and disease. Hum Genet. 2016;135:851–67. doi:10.1007/s00439-016-1683-5.

11. Masaki K, Sonobe Y, Ghadge G, Pytel P, Lépine P, Pernin F, et al. RNA-binding protein altered expression and mislocalization in MS. Neurol Neuroimmunol Neuroinflamm 2020;7:e704. doi:10.1212/NXI.0000000000000704.
12. Fabregat A, Sidiropoulos K, Viteri G, Marin-Garcia P, Ping P, Stein L, et al. Reactome diagram viewer: data structures and strategies to boost performance. Bioinformatics. 2018;34:1208–14. doi:10.1093/bioinformatics/btx752.
13. Hecker M, Fitzner B, Putscher E, Schwartz M, Winkelmann A, Meister S, et al. Implication of genetic variants in primary microRNA processing sites in the risk of multiple sclerosis. EBioMedicine. 2022;80:104052. doi:10.1016/j.ebiom.2022.104052.
14. Ritchie ME, Phipson B, Di Wu, Hu Y, Law CW, Shi W, Smyth GK. limma powers differential expression analyses for RNA-sequencing and microarray studies. Nucleic Acids Res. 2015;43:e47. doi:10.1093/nar/gkv007.
15. McCall MN, McMurray HR, Land H, Almudevar A. On non-detects in qPCR data. Bioinformatics. 2014;30:2310–6. doi:10.1093/bioinformatics/btu239.
16. Kent WJ, Sugnet CW, Furey TS, Roskin KM, Pringle TH, Zahler AM, Haussler D. The human genome browser at UCSC. Genome Res. 2002;12:996–1006. doi:10.1101/gr.229102.
17. Birnboim HC, Doly J. A rapid alkaline extraction procedure for screening recombinant plasmid DNA. Nucleic Acids Res. 1979;7:1513–23. doi:10.1093/nar/7.6.1513.
18. Friess J, Hecker M, Roch L, Koczan D, Fitzner B, Angerer IC, et al. Fingolimod alters the transcriptome profile of circulating CD4+ cells in multiple sclerosis. Sci Rep. 2017;7:42087. doi:10.1038/srep42087.
19. Roch L, Hecker M, Friess J, Angerer IC, Koczan D, Fitzner B, et al. High-Resolution Expression Profiling of Peripheral Blood CD8+ Cells in Patients with Multiple Sclerosis Displays Fingolimod-Induced Immune Cell Redistribution. Mol Neurobiol. 2017;54:5511–25. doi:10.1007/s12035-016-0075-0.
20. Angerer IC, Hecker M, Koczan D, Roch L, Friess J, Rüge A, et al. Transcriptome profiling of peripheral blood immune cell populations in multiple sclerosis patients before and

during treatment with a sphingosine-1-phosphate receptor modulator. CNS Neurosci Ther. 2018;24:193–201. doi:10.1111/cns.12793.

21. Hoffman RW, Merrill JT, Alarcón-Riquelme MME, Petri M, Dow ER, Nantz E, et al. Gene Expression and Pharmacodynamic Changes in 1,760 Systemic Lupus Erythematosus Patients From Two Phase III Trials of BAFF Blockade With Tabalumab. Arthritis Rheumatol. 2017;69:643–54. doi:10.1002/art.39950.
22. Li Y, Higgs RE, Hoffman RW, Dow ER, Liu X, Petri M, et al. A Bayesian gene network reveals insight into the JAK-STAT pathway in systemic lupus erythematosus. PLoS One. 2019;14:e0225651. doi:10.1371/journal.pone.0225651.
23. Scott A, Petrykowska HM, Hefferon T, Gotea V, Elnitski L. Functional analysis of synonymous substitutions predicted to affect splicing of the CFTR gene. J Cyst Fibros. 2012;11:511–7. doi:10.1016/j.jcf.2012.04.009.
24. Untergasser A, Nijveen H, Rao X, Bisseling T, Geurts R, Leunissen JAM. Primer3Plus, an enhanced web interface to Primer3. Nucleic Acids Res. 2007;35:W71–74. doi:10.1093/nar/gkm306.
